# RRNPP quorum-sensing repertoires in the salivarius group genomes: overrepresentation and synchronous activation of SHP/Rgg systems in *Streptococcus thermophilus*

**DOI:** 10.1128/jb.00231-25

**Published:** 2025-08-26

**Authors:** Quentin Caillot, Alain Guillot, Thomas Lacroix, Lydie Oliveira-Correia, Eugénie Huillet, Gwénaëlle André, Pierre Nicolas, Rozenn Gardan

**Affiliations:** 1 Paris-Saclay university, INRAE, AgroParisTech, Micalis Institute 74288 , Jouy-en-Josas, France; 2 Paris-Saclay university, INRAE, AgroParisTech, MaIAGE, Jouy-en-Josas, France; University of Illinois Chicago, Chicago, Illinois, USA

**Keywords:** *Streptococcus thermophilus*, quorum sensing, RRNPP, Rgg, ComR, SHP, signaling peptide, RaS-RiPPs

## Abstract

In Bacillota, quorum sensing can be mediated by RRNPP regulators that are activated by autoinducing peptides (AIPs). In this study, we derived a hidden Markov model profile from a 3D-informed alignment to establish RRNPP repertoires for 527 genomes of streptococci in the salivarius group and identified probable AIPs. The salivarius group encompasses *Streptococcus salivarius* and *Streptococcus vestibularis*, which are part of the normal human oral microflora, and *Streptococcus thermophilus*, one of the most widely used bacteria in the dairy industry. We observed a large amount of plasticity in these repertoires, as well as profound differences among species. Notably, *S. salivarius* displayed an accumulation of ComR regulators, while *S. thermophilus* displayed an accumulation of Rgg regulators. The latter family included SHP-associated Rgg regulators, systems in which SHPs serve as AIPs; most of these regulators control the production of post-translationally modified peptides (RaS-RiPPs). Their level of richness contrasts with the genome reduction that accompanied *S. thermophilus*’ adaptation to milk. We then used liquid chromatography-high resolution tandem mass spectrometry to analyze the activity of the eight most common SHP/Rgg systems by characterizing the SHPs and RaS-RiPPs found in the supernatants. We detected four SHPs and one RaS-RiPP that have never been seen before in *S. thermophilus*, and we showed that seven of the eight SHP/Rgg systems were functional. Finally, by simultaneously monitoring the amounts of both the SHPs and RaS-RiPPs, we demonstrated that the fates of these two peptide types differed during growth. SHP presence in the supernatant was transient, a pattern likely related to the peptides’ signaling role.

**IMPORTANCE:**

*Streptococcus thermophilus* possesses an unusually high number of Rgg regulators, which are activated by SHP pheromones that control the production of RaS-RiPPs, peptides with cyclization motifs and growth inhibition properties. We conducted an *in silico* analysis of regulator repertoires across a wide range of strains; a subsequent experimental study revealed that the majority of the SHP/Rgg systems were functional. Employing an optimized liquid chromatography-high resolution tandem mass spectrometry protocol, we were able to better detect and follow SHP and RaS-RiPP accumulation. While RaS-RiPPs accumulated during growth, SHPs were only transiently present in the extracellular environment. This observation suggests that we could manipulate quorum sensing by adding SHPs to the growth medium and highlights the need to study the functions of the RaS-RiPPs.

## INTRODUCTION

In bacteria, quorum sensing (QS) is a process where signaling molecules are exported into the extracellular medium, eliciting responses that allow gene expression to track cell density and give rise to group-level behavior. In Bacillota, these signaling molecules are mainly auto-inducing peptides (AIPs). The expression of AIP-encoding genes is regulated by QS, creating a positive feedback loop. AIPs are recognized either extracellularly by histidine kinases of two-component systems or intracellularly by regulators of the RRNPP superfamily (1). The superfamily is named for its prototypical members, Rap, Rgg, NprR, PlcR, and PrgX, which represent its constitutive families. Structural studies characterizing at least one member of each family have shown that the regulators display similarities in 3D topology (2). At their C-terminus, these regulators have tetratricopeptide repeat (TPR) domains capable of binding their cognate AIPs, which are often encoded by a neighboring gene. Following complexation with their AIPs, regulators undergo family-specific structural changes and become active (3–7). At their N-terminus, the transcriptional regulators (i.e*.,* all the regulators except the Rap phosphatases) have a helix-turn-helix (HTH) domain that is responsible for DNA binding.

The group-level behaviors controlled by RRNPP regulators are diverse. PrgX is a regulator that controls plasmid transfer in enterococci (8); this family works with activator and inhibitor peptides. PlcR regulators control virulence in the *B. cereus sensu lato* group via their PapR AIPs (9). They are also involved in virulence and commensalism in *Streptococcus pneumoniae* (10), where they are called TprA regulators; we will use this designation hereafter since this study focuses on streptococci (11). Rap phosphatases regulate sporulation, competence, and the production of degradative enzymes in bacilli via their Phr AIPs (12). NprR is a dual phosphatase/transcriptional regulator that helps control necrotrophic behavior in *B. cereus sensu lato* via NprX AIPs (9).

The Rgg family is likely the most diverse and can be divided into several subfamilies based on phylogeny and AIP amino acid sequences (13). One subfamily comprises the Rgg regulators associated with AIPs called short hydrophobic peptides (SHPs) (14). These regulators are involved in biofilm formation, lysozyme resistance, and immunosuppressive activities in *Streptococcus pyogenes* (15–17), virulence in *Streptococcus agalactiae* (18), environmental adaptation and colonization in *S. pneumoniae* (10), and the production of secreted cyclic peptides in streptococci in general (19, 20). All the SHP/Rgg systems described to date function in a similar way. First, the SHP precursor is synthesized and matured, usually in front of a glutamate or aspartate residue, making it easier to predict the mature SHP’s sequence. This mature peptide is then exported into the extracellular medium and imported by an oligopeptide permease. Once inside the cell, mature SHPs can interact directly with Rgg transcriptional regulators, which are subsequently activated (2). Another subfamily comprises the RopB regulators associated with AIPs called leaderless communication peptides (LCPs) (13, 21). RopB regulators are involved in virulence in *S. pyogenes*. It is worth noting that some *rgg* genes do not seem to have an AIP-encoding gene in their immediate neighborhood. Such stand-alone Rgg regulators may function without AIPs or via AIPs encoded elsewhere. For instance, no AIP has yet been found for the regulator of the glucosyltransferase gene in *Streptococcus gordonii*, the regulator for which the Rgg family is named (22). The RRNPP superfamily encompasses other families as well. These include the family of AimR regulators (whose AIPs are AimPs), which control whether lysis proceeds in temperate phages of bacilli (23), as well as the family of ComR regulators (whose AIPs are XIPs, the active mature forms of the inactive precursors ComS), which are involved in competence during natural transformation in certain groups of streptococci (24). This list is not exhaustive, as research has recently identified other members of the RRNPP superfamily and their associated AIPs (25).

The salivarius group consists of three species: *Streptococcus salivarius*, *Streptococcus vestibularis*, and *Streptococcus thermophilus*. Both *S. salivarius* and *S. vestibularis* belong to the normal human oral microflora, and they sometimes cause opportunistic infections. They are genetically similar to *S. thermophilus,* but their genomes are about 20% larger (26, 27). *S. thermophilus* is one of the most widely used bacterial species in the dairy industry. It is essential in yogurt production and is frequently used as a starter in cheese making. It thrives in dairy-processing environments, and its adaptation to life in milk has been accompanied by the loss of many genes (28, 29). In parallel with this regressive evolution, *S. thermophilus* has acquired numerous small genomic islands via horizontal gene transfer, including regions that encode RRNPP regulators. For example, the genome of *S. thermophilus* strain LMD-9 contains five SHP/Rgg loci (19).

*S thermophilus* has become a model for studying SHP/Rgg systems due to its diverse repertoire of the latter (19, 30, 31). Most of its SHP/Rgg systems appear to control the transcription of proximal operons involved in the production of ribosomally synthesized peptides that are post-translationally modified by radical SAM enzymes (RaS-RiPPs) (32). RaS-RiPPs are scientifically intriguing because of their unique cyclization motifs (20). Researchers have observed interesting growth inhibition activities for two of them, streptosactin and bicyclostreptin, in *S. thermophilus*. Given their narrow spectra, streptosactin and bicyclostreptin are suspected to be involved in fratricide and growth regulation, respectively (33, 34). Antimicrobial activity has been observed with other RaS-RiPPs, such as darobactin. However, the functions of most of these compounds are unknown and likely vary (35).

In this study, we improved the current understanding of the RRNPP regulator repertoire in salivarius-group streptococci; to this end, we used a dedicated bioinformatics approach and a large set of genomes. We showed that the group’s three members differ in their distribution and prevalence of RRNPP regulators and that *S. thermophilus* strains have accumulated *rgg* genes, particularly *shp*/*rgg* genes, in their genomes. We then focused on SHP/Rgg systems. As *shp* genes are part of the SHP/Rgg regulon, we used liquid chromatography-high resolution tandem mass spectrometry (LC-HR-MS/MS) to confirm the operation of these systems by assessing mature SHP presence in the culture supernatant of selected strains. We also assessed the presence of RaS-RiPPs. We found that seven of the eight most common SHP/Rgg systems in *S. thermophilus* are functional. Among these systems were four that had not yet been studied experimentally. Finally, our simultaneous assessments of mature SHP and RaS-RiPP quantities in the supernatant revealed that these two peptide types differ in their fates during growth.

## RESULTS

### *In silico* identification of RRNPP regulators in salivarius-group streptococci

**Fig 1:**
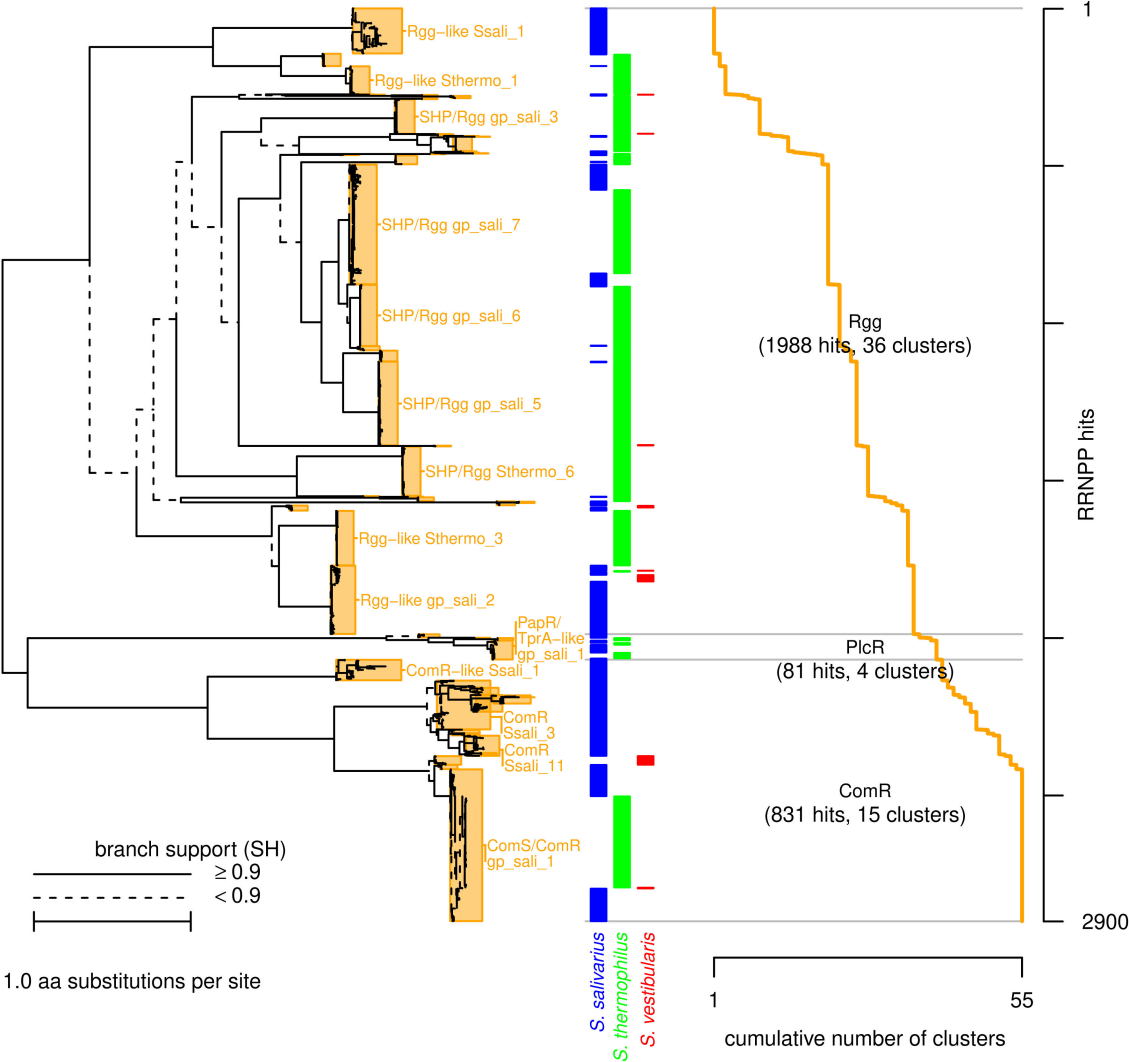
Phylogenetic tree, classification, and clustering of RRNPP regulators found in the group salivarius. The phylogenetic tree, which was reconstructed using an HMM alignment of 2,900 protein sequences collected from 527 genomes, is shown on the left, with an indication of the level of statistical support of each branch (SH-test). The orange rectangles surrounding the leaves of the tree represent clusters defined by a cutoff of 90% pairwise identity. Labels are reported for the clusters containing at least 50 sequences. The bacterial species in which the regulators were found are indicated by colored polygons, with three colors representing the three species: *S. salivarius*, *S. thermophilus*, and *S. vestibularis*. Horizontal gray lines separate the three families: ComR, PlcR, and Rgg. The cumulative number of clusters as new RRNPP regulators are added, according to their position in the tree, is shown by the orange line on the right side of the plot. RRNPP regulators are ordered in Table S2 according to their positions in the tree (the *y*-axis coordinate indicates the table position).

We characterized the repertoire of RRNPP regulators in salivarius-group streptococci using a search based on a single hidden Markov model (HMM) profile. This approach differs from others that relied on individual HMM profiles for each family of regulators (36), focused on distinct families using BLAST analysis (2, 13, 37), or employed more complex combinations of clues, including colocalization with putative AIPs (25). The single HMM profile was created from a 3D-informed alignment of 33 sequences belonging to the Rgg, ComR, PlcR, and PrgX families (Table S1). The advantage of using this single profile is that it produced a unique alignment and set of results, which were constructed independently of the AIPs, as their corresponding genes may be located elsewhere in the genome. The bioinformatic workflow, applied to 291 *S*. *thermophilus* genomes, 204 *S*. *salivarius* genomes, and 32 *S*. *vestibularis* genomes, yielded a total of 2,900 hits that gave rise to 55 RRNPP clusters displaying more than 90% pairwise identity (Table S2). Using information from the baits, these clusters could be assigned to the families PlcR (81 hits), ComR (831 hits), and Rgg (1,988 hits), which could be further subdivided into subfamilies with the signifier “like” indicating less than 40% shared identity (Table 1). We manually scanned predicted short coding sequences (CDSs) upstream and downstream of the RRNPP genes to identify genes encoding peptides with probable AIP functions. When found, the name of the precursor AIP (e.g., SHP and ComS) was included in the RRNPP cluster identifier (Table S2). To clarify the evolutionary relationship between these RRNPP genes, we constructed a phylogenetic tree for the RRNPPs of the entire salivarius group (Fig. 1, see also Fig. S1 to S3 for the separate trees for the three species).

**TABLE 1:** Nomenclature for the RRNPP clusters identified in the salivarius group^*c*^

Family	Subfamily^*a*^^,*b*,*c*^	AIP precursor	AIP
PlcR	PapR/TprA	PapR	Mature PapR
	PapR/TprA-like	PapR	Mature PapR
ComR	ComS/ComR	ComS	XIP
	ComR	Unknown	Unknown
	ComR-like	Unknown	Unknown
Rgg	SHP/Rgg	SHP	Mature SHP
	SHP/Rgg-like	SHP	Mature SHP
	LCP/RopB	LCP	LCP
	MutS/MutR	MutS	MSP
	Rgg	Unknown	Unknown
	Rgg-like	Unknown	Unknown

^*a*^ The signifier “like” indicates less than 40% shared identity with one of the baits in Table S1.

^*b*^ The Rgg subfamily refers to either an Rgg with more than 40% shared identity with the *Streptococcus gordonii* bait Rgg or an SHP/Rgg bait for which an AIP has not been detected.

^*c*^ In relation to the ComS/ComR family, the ComR and ComR-like subfamilies are referred to as “extra-ComR” in the text.

**Fig 2:**
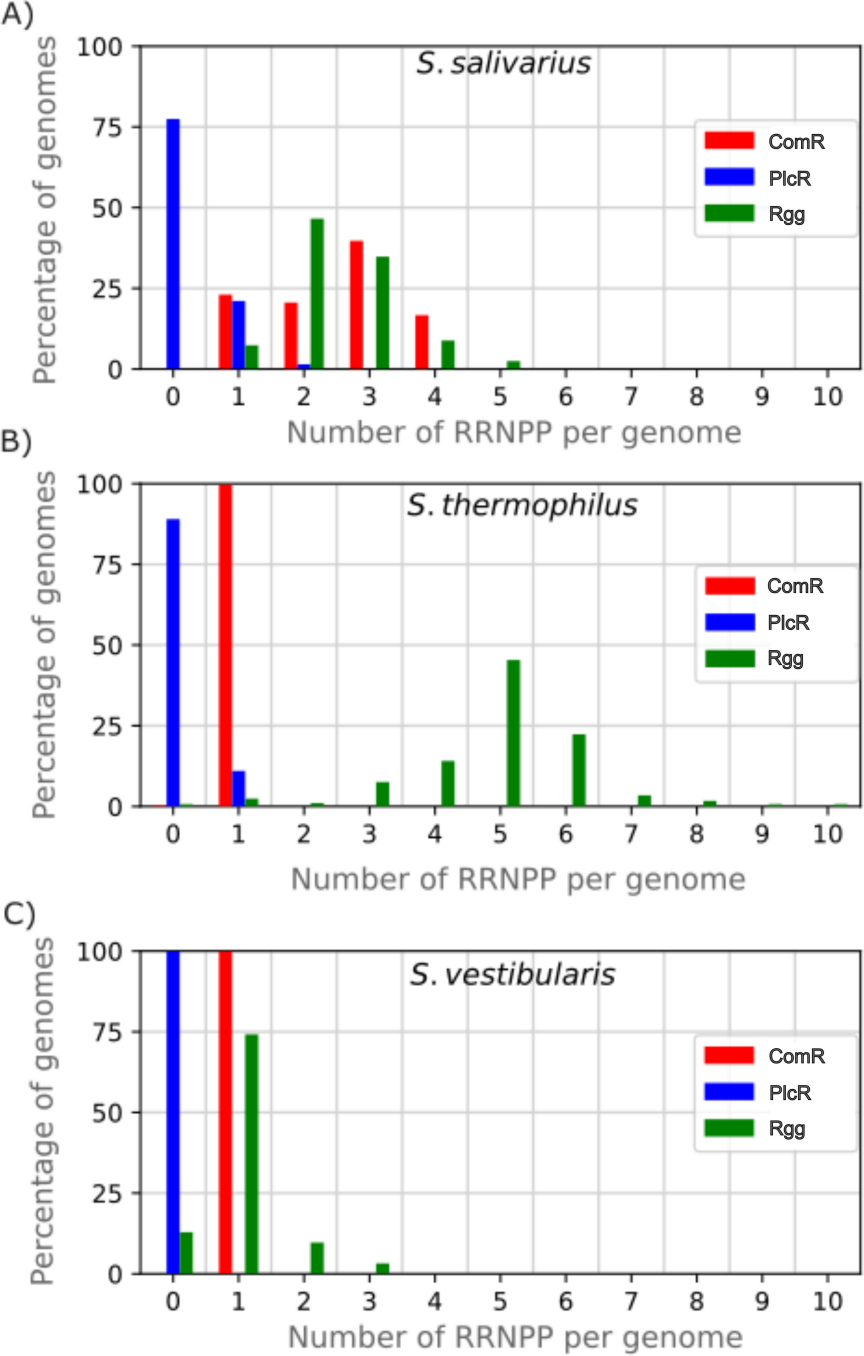
Percentage of genomes with different numbers of RRNPP clusters in the group salivarius. The ComR, PlcR, and Rgg systems are shown in red, blue, and green, respectively, for *S. salivarius* (**A**), *S. thermophilus* (**B**), and *S. vestibularis* (**C**).

The distribution of RRNPP gene number per genome differed significantly among the three RRNPP families and the three species of the salivarius group (Fig. 2). Representatives of the PlcR family were observed in a smaller percentage of *S. thermophilus* genomes (22%) and *S. salivarius* genomes (10%); generally, there was only one TprA regulator encoded per genome, although a few *S. salivarius* genomes (3/204) encoded two TprA regulators. None of the *S. vestibularis* genomes encoded a TprA regulator, but the species was represented by only 32 genomes. Broader sampling efforts could reveal the presence of genes encoding TprA in other isolates. All strains of the three species encoded a representative of the ComS/ComR subfamily involved in controlling competence. This was the only ComR regulator encoded in *S. thermophilus* and *S. vestibularis* isolates. The only exception was one *S*. *vestibularis* genome that had been fragmented into 385 contigs, where the gene may have been missed. In contrast to the two other species, in *S. salivarius*, there is encoding of extra ComR regulators (mean = 2.5 *comR* genes per genome, including ComS-associated ComR regulators). Examination of the predicted short CDSs led to the identification of any obvious AIP candidates for the extra ComR regulators. In all three species, the Rgg family was the best represented of the RRNPP superfamily, and the number of genes encoding Rgg representatives per genome varied among isolates. Nevertheless, the three species exhibited marked differences in their per-genome numbers of genes encoding Rgg representatives: they were much higher in *S. thermophilus* (mean = 4.9 genes per genome) than in *S. vestibularis* (mean = 1.0 genes per genome) and *S. salivarius* (mean = 2.5 genes per genome).

### Diversity and plasticity of the SHP/Rgg system repertoire

**Fig 3:**
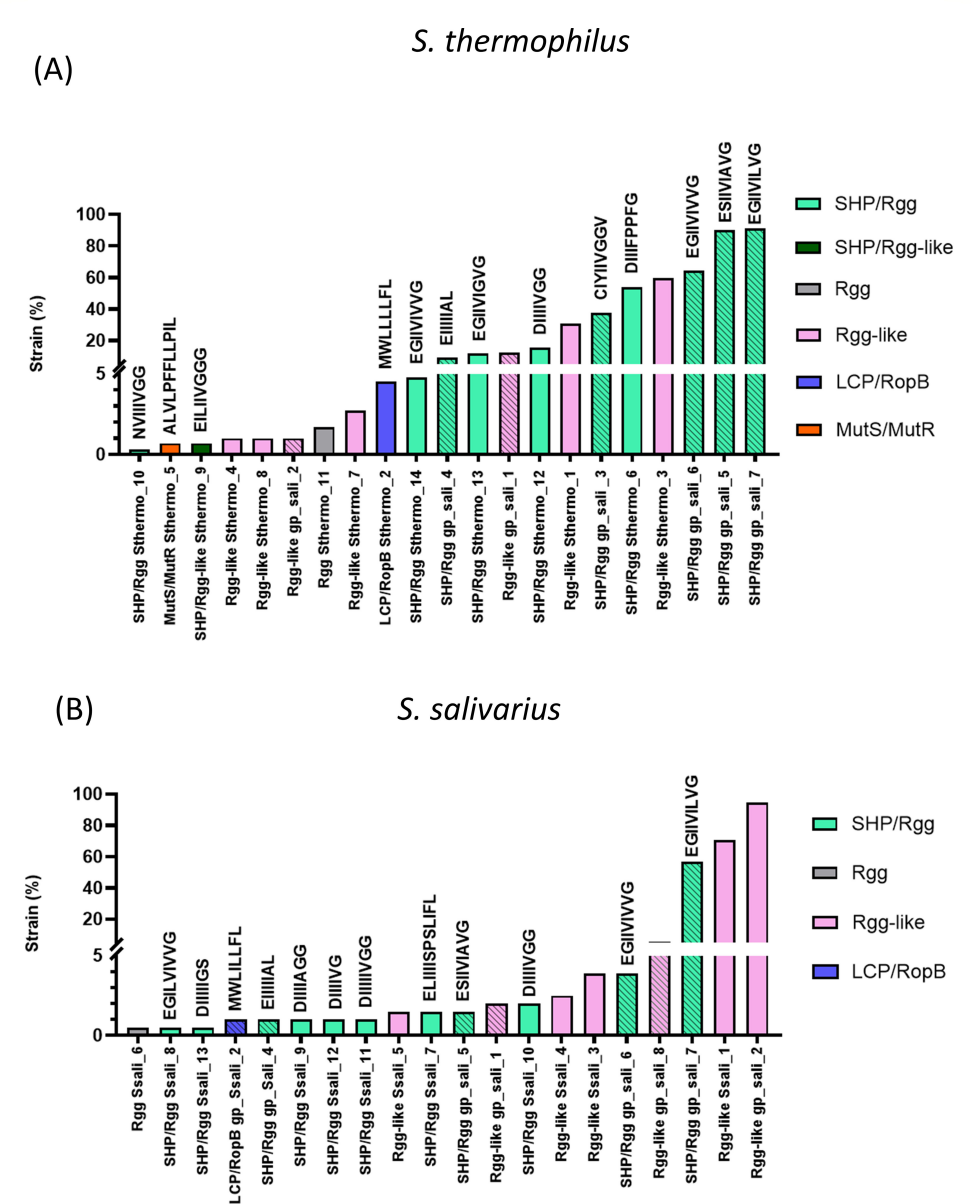
Percentage of strain with one Rgg of each cluster in *S. thermophilus* (**A**) and *S. salivarius* (**B**). Each color refers to an RRNPP subfamily, and the hatched colors indicate clusters that are shared within the salivarius group. Putative mature forms of peptides associated with each RRNPP cluster are mentioned.

**Fig 4:**
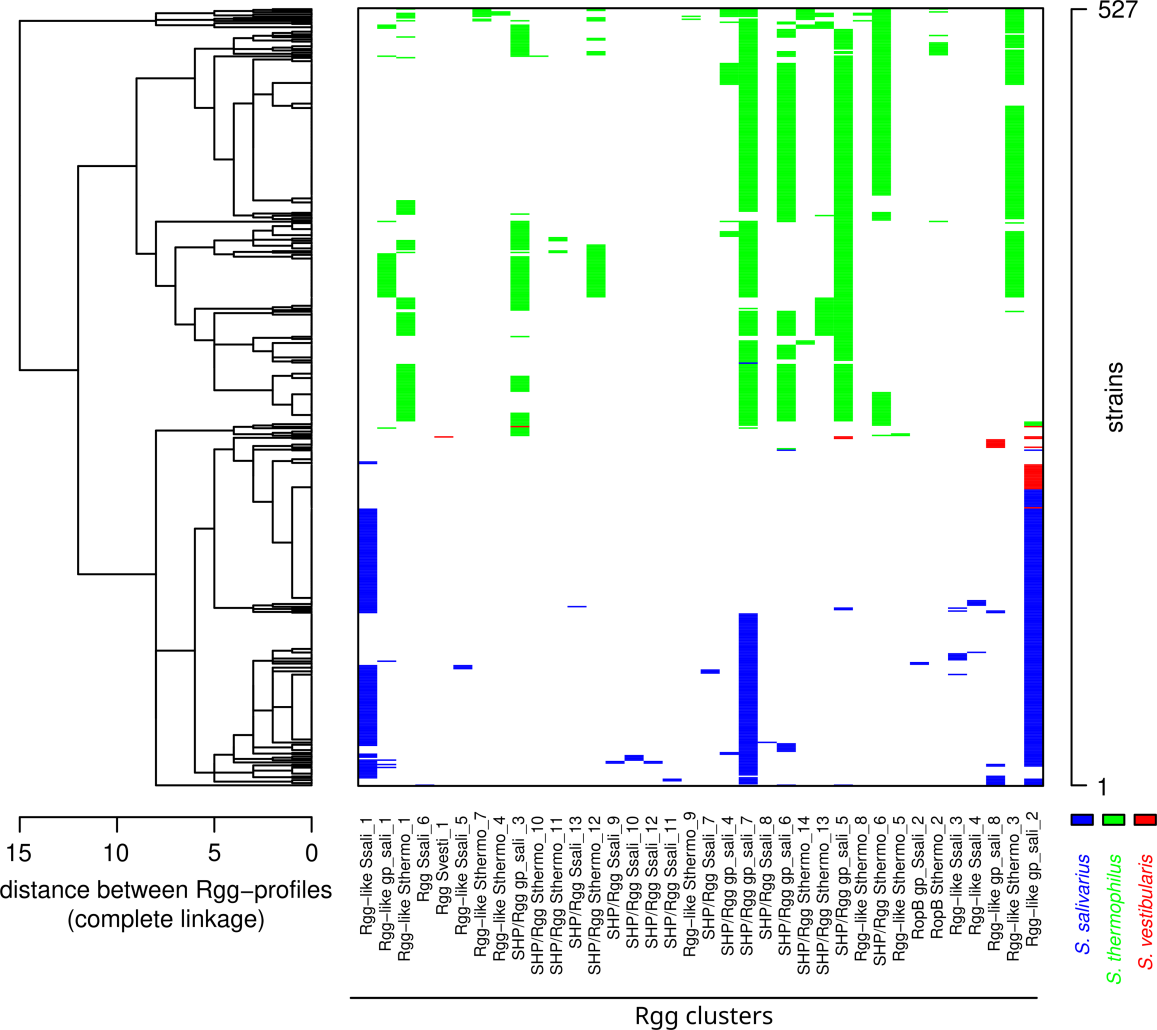
Heatmap representation of the Rgg profiles found in the salivarius group. The 527 strains (rows) are ordered vertically according to the hierarchical clustering tree on the left side of the plot. In this tree, the distance between two profiles is defined as the number of differences in the vector of presence/absence of the 36 Rgg clusters (columns). The three colors in the heatmap distinguish the three species: *S. thermophilus*, *S. salivarius,* and *S. vestibularis*.

The Rgg family was the best represented and most diverse of the RRNPP families found to be encoded in the salivarius-group species: based on the threshold of 90% pairwise identity, we observed a total of 36 clusters for the Rgg family compared to 15 clusters for the ComR family and 4 clusters for the PlcR family. Out of the 36 Rgg clusters, 21 were found in *S. thermophilus* (Table 2), 20 were found in *S. salivarius* (Table S3), and 6 were found in both species (Fig. 3). Five clusters were found in *S. vestibularis* (Table S4), of which only one was species specific. Within each species, cluster prevalence varied widely; while some clusters were represented in only one strain, other clusters were represented in a majority of strains. Five clusters occurred in more than 50% of *S. thermophilus* strains, three clusters occurred in more than 50% of *S. salivarius* strains, and no clusters reached this level of prevalence in *S. vestibularis* (Fig. 3; Fig. S4). To explore Rgg profile plasticity, we represented the occurrence of each Rgg cluster across the salivarius-group strains using a heatmap (Fig. 4). The strains of *S. thermophilus* were associated with 69 different Rgg occurrence profiles, compared with 38 and 5 in the strains of *S. salivarius* and *S. vestibularis*, respectively. The four most abundant profiles represented 41.2% of the strains in *S. thermophilus* compared to 71.1% of the strains in *S. salivarius* (none of the 34 other profiles was represented by more than 5/204 strains). The profiles of two randomly selected strains differed, on average, by 3.9 Rgg clusters for *S. thermophilus*, 1.6 Rgg clusters for *S. salivarius*, and 1.0 Rgg clusters for *S. vestibularis*. These results indicate that considerable plasticity exists in the repertoire of Rgg regulators, particularly in *S. thermophilus*, which displayed the greatest abundance of Rgg regulators and the greatest diversity of Rgg profiles among strains.

**TABLE 2:** RRNPP clusters in *Streptococcus thermophilus*

RRNPP family	RRNPP cluster identifier^*a*^	Putative AIP^*b*^	Strain/isolate^*c*^	Blastp best bait identifier	% identity (coverage)
ComR	ComS/ComR_gp_sali_1_ (290)	MKTLKIFVLFSLL**IAILPYFAGCL** (155)	TH1477	bait16_ComRSt	98 (100)
		MKTLKIFVLFSLLI*P*ILPYFAGCL (130)	ST057-1	bait16_ComRSt	99 (100)
		MKTLKIFVLF*P*LLIAILPYFAGCL (2)	TSGB 4234	bait16_ComRSt	98(100
		MKTLKIFVLFSLLIAI*F*PYFAGCL (2)	Vach60	bait16_ComRSt	100 (100)
PlcR	PapR/TprA-like_Sthermo_1_ (8)	MRKFLKKLIVISLLGGLILAIGKGGTNKVKPMNGIDPGPANIMVK (8)	JIM8232	bait28_TprA	37 (97)
PlcR	PapR/TprA-like_gp_sali_1_ (24)	MKKRLKKLTAICLLSFLLISFGIGTDSGVKTSGRIDPGPAHSMVS (4)	TSGB 4234	bait28_TprA	40 (98)
		MKK*G*LKKLTAICLLSFLLISFGIGTDSGVKTSGRIDPGPAHSMVS (1)	4067	bait28_TprA	40 (98)
Rgg	Rgg-like_gp_sali_1_ (36)	N.D.^*d*^ (36)	LMG 18311	bait8_SHP/Rgg2Sd	28 (66)
Rgg	Rgg-like_Sthermo_1_ (89)	N.D.	DSM 20617	bait27_Rgg	26 (88)
			Roque89	bait8_SHP/Rgg2Sd	26 (82)
			252	bait7_SHP/Rgg2Spy	26 (88)
Rgg	LCP/RopB_Sthermo_2_ (13)	MWLLLLFL (10)	stT-1	bait10_LCP/RopB	54 (97)
Rgg	Rgg-like_gp_sali_2_ (3)	N.D.	TSGB 4239	bait8_SHP/Rgg2Sd	34 (96)
Rgg	Rgg-like_Sthermo_3_ (174)	N.D.	LMG 18311	bait5_SHP/RovS	33 (92)
Rgg	Rgg-like_Sthermo_4_ (3)	N.D.	stT-1	bait27_Rgg	30 (97)
Rgg	MutS/MutR_Sthermo_5_ (2)	MLKTILKRIISLALVLPFFLLPIL (2)	STH_CIRM_956	bait31_MutR	61 (98)
Rgg	SHP/Rgg_Sthermo_6_ (157)	MKKVIAIFLFIQTVVVI**DIIIFPPFG** (156)	ST13	bait1_SHP/RggSt1299	100 (100)
Rgg	Rgg-like_Sthermo_7_ (8)	N.D.	252	bait27_Rgg	34(95)
Rgg	Rgg-like_Sthermo_8_ (3)	N.D.	252	bait3_SHP/RggSmu1509	31 (97)
Rgg	SHP/Rgg-like_Sthermo_9_ (2)	MSDLKFCKLLFLLTLFEILIIVGGG (2)	st324	bait4_SHP/RggSt0182	31 (98)
Rgg	SHP/Rgg_Sthermo_10_ (1)	MKKFLKYSLVILANVIIIVGG (1)	8253 (205)	bait6_SHP/Rgg3Spy	59 (99)
Rgg	Rgg_Sthermo_11_ (5)	N.D.	NBRC 13957	bait13_SHP/RggSmi0094	73 (100)
Rgg	SHP/Rgg_Sthermo_12_ (46)	MEKVSKILPILILVMDIIIIVGG (45)	STH_CIRM_1047	bait9_SHP/Rgg3St	100 (100)
		M*K*K*T*SK*F*LPILILVMDIIIIVGG (1)	252	bait9_SHP/Rgg3St	99 (100)
Rgg	SHP/Rgg_gp_sali _3_ (109)	MKLLKIIVLLTCIYIIVGGV (92)	STH_CIRM_1125	bait4_SHP/RggSt0182	100 (100)
		MKLLKIIVLLTCIY*T*IVGGV (16)	CNRZ1066	bait4_SHP/RggSt0182	100 (100)
		M*E*LLKIIVLLTCIYIIVGGV (1)	ena-SAMPLE-787-33427	bait4_SHP/RggSt0182	100 (100)
Rgg	SHP/Rgg_gp_sali_4_ (27)	MNISIKRFLMILLEIIIIIAL (25)	STH_CIRM_1121	bait3_SHP/RggSmu1509	47 (98)
		MNI*F*IKRFLMILLEIIIIIAL (2)	MV-FAST4	bait3_SHP/RggSmu1509	48 (98)
Rgg	SHP/Rgg_Sthermo_13_ (35)	MNKKALFSLLFVIL**EGIIVIGVG** (34)	TSGB 4234	bait2_SHP/RggSt1358	63 (98)
Rgg	SHP/Rgg_gp_sali_5_ (262)	MNKESFLAILLLIFESIIVIAVG (262)	ACA-DC 2	bait2_SHP/RggSt1358	63 (99)
Rgg	SHP/Rgg_gp_sali_6_ (187)	MKKQILLTLLLVVF**EGIIVIVVG** (184)	St-10	bait2_SHP/RggSt1358	97 (100)
		MKKQILLTLLLVVFE*D*IIVIVVG (2)	Vach60	bait2_SHP/RggSt1358	100 (100)
Rgg	SHP/Rgg_Sthermo_14_ (14)	MKKQILLTLLLVVFEGIIVIVVG (6)	TSGB 4141	bait2_SHP/RggSt1358	88 (100)
		MK*N*Q*K*LL*P*LL*FLL*FEGIIIIVVG (8)	TSGB 4234	bait2_SHP/RggSt1358	88 (100)
Rgg	SHP/Rgg_gp_sali_7_ (265)	MKKQKLLLLVVLVCEGIIVILVG (263)	TSGB 4236	bait2_SHP/RggSt1358	81 (98)
		MKK*P*KLLLLVVLVCEGIIVILVG (1)	TSGB 4235	bait2_SHP/RggSt1358	81 (98)
		MKKQKLLLLVVLVCEGVIVILVG (1)	st263	bait2_SHP/RggSt1358	81 (98)

^*a*^ The hit number is in parentheses.

^*b*^ Boldface type indicates the sequence of a mature form of the AIP as observed experimentally in *S. thermophilus*. Mutations in comparison to the most common form are underlined and italicized.

^*c*^ One strain and its AIP (the first in the list in Table S2) were selected for each RRNPP cluster.

^*d*^ N.D., not detected (no peptide detected or no obvious AIP signature for a detected peptide).

Loci encoding SAM radical enzymes or ThiF enzymes were found in 11/21 of the regions surrounding the *rgg* genes in *S. thermophilus* (Table S5); this figure was 6/20 for *S. salivarius* (Table S6). These enzymes are known to be involved in the post-translational modification of peptides, suggesting that these loci encode products involved in the production of RiPPs. We also observed numerous fragments of transposase-encoding genes near the *rgg* genes in both species and near the extra *comR* genes in *S. salivarius* specifically (Table S2).

**Fig 5:**
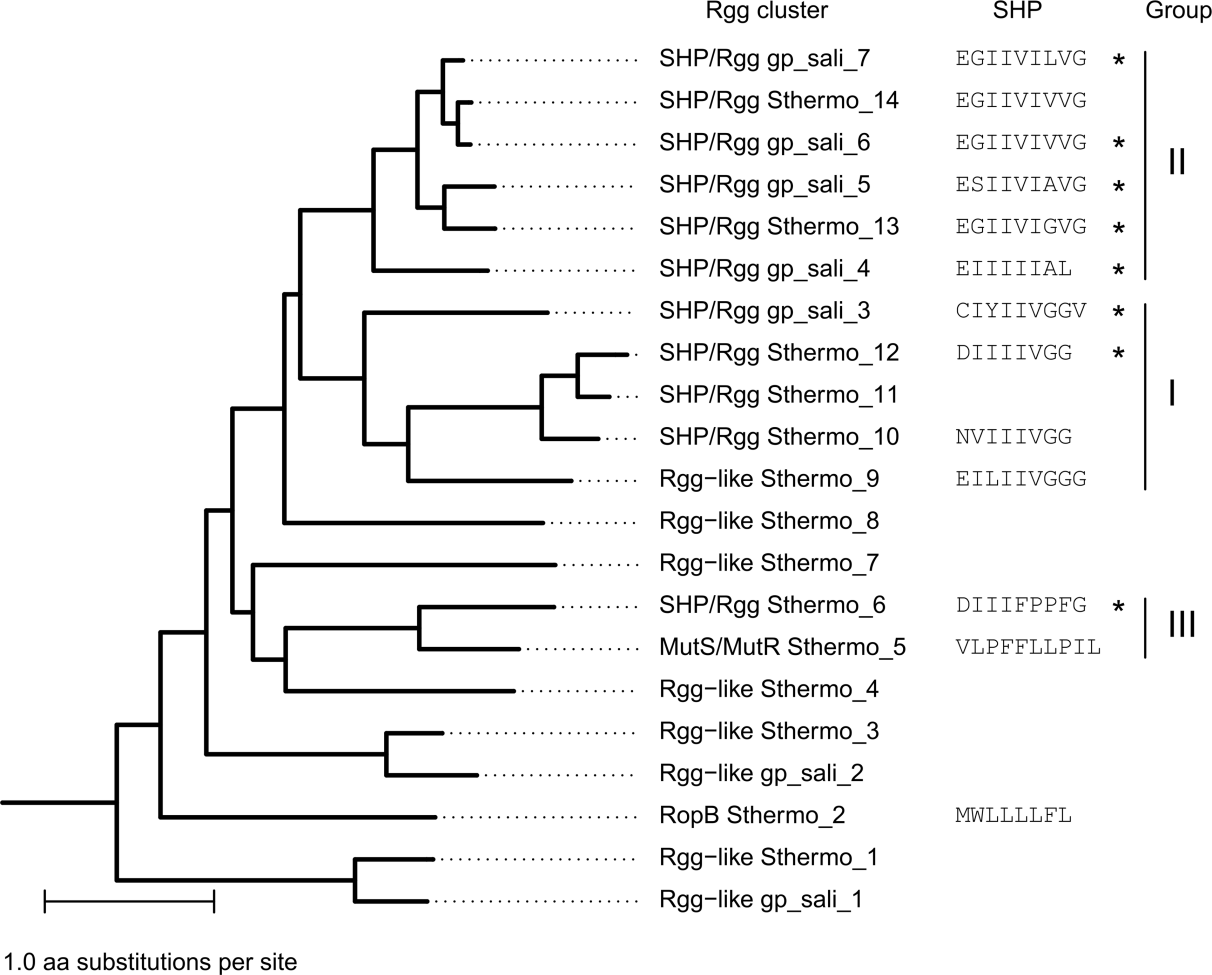
Phylogenetic tree of the Rgg clusters in *S. thermophilus*. One Rgg representative has been chosen for each cluster. The amino acid sequence of the mature SHPs of each cluster is annotated around the tree. * indicates that the peptide has been identified experimentally in this study. SHP/Rgg groups are annotated according to Table S2.

In *S. thermophilus*, 52% of the *rgg* clusters were found to belong to the SHP/Rgg subfamily; this figure was 55% for *S. salivarius* and 60% for *S. vestibularis* (Table 2; Tables S3 and S4). The predicted SHPs have 20–26 amino acids along with an N-terminal Lys (K) residue, a C-terminal stretch of hydrophobic residues, and, often, a C-terminal Gly (G) and/or a central acidic residue, Asp (D) or Glu (E). Three SHP/Rgg clusters were present in more than 50% of the strains (Fig. 3A). A phylogenetic tree of the *S. thermophilus* Rgg clusters was constructed using a representative from each cluster (Fig. 5). We classified the SHP/Rgg clusters in groups I, II, and III in accordance with a previously established nomenclature, taking into account the genetic orientation of the *shp* and *rgg* genes, as well as the SHP amino acid sequences (30). It is worth noting that the SHP/Rgg group III has recently been assigned to the MutRS peptide-based quorum-sensing system (37), which is discussed in more detail in the “Discussion” section.

### Mature SHPs encoded by *S. thermophilus* genomes are secreted during the mid-exponential growth phase

The *S. thermophilus* genome is undergoing a regressive process (29). To confirm the functionality of the eight major SHP/Rgg systems corresponding to the clusters found in more than 5% of the *S. thermophilus* strains, we assessed the presence of mature SHPs in the extracellular medium (Table 2; Fig. 3A). Indeed, *shp* gene expression is controlled by the SHP/Rgg complexes, creating a positive feedback loop. The targets usually also comprise an additional operon located in the vicinity of the Rgg-encoding gene (2). For this work, we selected the strains JIM8232 and CNRZ1066 to serve as representatives. We detected the presence of four new mature SHPs: SHP_gp_sali_5_ in both strains; SHP_gp_sali_4_ in strain JIM8232; and SHP_Sthermo_12_ and SHP_gp_sali_3_ in strain CNRZ1066 (Table 3; Fig. S5 to S8). The latter mature SHP was only observed after we reduced and alkylated the crude supernatant to stabilize the thiol group of the cysteine (Table 3; Fig. S8). We also detected the three known mature SHPs (Table 3; Fig. S9 to S11): SHP_Sthermo_6_, SHP_gp_sali_6_, and SHP_Sthermo_13_ (32). However, we did not detect SHP_gp_sali_7_, whose locus was observed in both strains. To confirm these results, all retention times and mass spectra for the above mature SHPs were compared to those for synthetic SHPs injected into the mass spectrometer under the same conditions (Fig. S5 to S11; Tables S7 to S13). Since the retention time for the synthetic SHP_gp_sali_7_ was similar to that of the other SHPs (Fig. S12; Table 3), our failure to detect mature SHP_gp_sali_7_ in the crude supernatant likely reflects its absence or its presence at very low concentrations.

**TABLE 3:** Detection of natural SHPs by LC-HR-MS/MS in the raw supernatant of *S. thermophilus* strains JIM8232 and CNRZ1066^*a*^^,*b*^

Peptide	Mature sequence	Gravy Index	RT^*a*^ of synthetic standard	JIM8232^*b*^^,*c*^		CNRZ1066^*b*^^,*c*^	
				RT	EIC^*d*^	RT	EIC^*d*^
SHP_gp_sali_5_	ESIIVIAVG	2.11	25.3	25.3	6.13E+05	25.3	1.3E+06
SHP_Sthermo_6_	DIIIFPPFG	1.33	29.8	29.8	6.8E+05		
SHP_gp_sali_6_	EGIIVIVVG	1.91	26.2	26.2	1.8E+06		
SHP_gp_sali_7_	EGIIVILVG	2.38	26.0	N.D	N.D	N.D	N.D
SHP_Sthermo_13_	EGIIVIGVG	1.91	25.2	25.1	3.04E+06	25.1	1.73E+05
SHP_gp_sali_4_	EIIIIIAL	3.08	28.6	28.6	9.5E+04		
SHP_gp_sali_3_	CIYTIVGGV	1.9	24.7			24.6	2.5E+07
SHP_Sthermo_12_	DIIIIVGG	2.24	26.4			26.3	2.0E+05

^*a*^ RT, retention time (min).

^*b*^ N.D, not detected.

^*c*^ Gray, the SHP encoding gene is not present in the genome.

^*d*^ Extracted ion chromatogram, area under the curve of the ion current signal.

To confirm that these results could be generalized to the species level in *S. thermophilus*, we also looked for the eight mature SHPs in three other strains: the reference starter LMD-9, the industrial starter N4L, and CIRM30. The latter strain was selected because it carries the SHP/Rgg_gp_sali_7_ locus with a complete RaS-RiPP target operon, which encodes a peptide, a radical SAM enzyme, and a transporter. This complete operon found in *Streptococcus suis* allows the production of a RaS-RiPP called ryptide (38). In the other *S. thermophilus* strains included in our *in silico* study (264/265), the target operon only encoded the transporter. As shown in Tables S14 and S15, all the mature SHPs were detected; the exceptions were SHP_gp_sali_7_, which was not seen in any of the three strains, and SHP_Sthermo_6_, which was not seen in strain N4L.

We then manually obtained the extracted ion current (EIC) signals of the monocharged mature SHP to compare the amount of each peptide in the supernatants of the different strains. We observed that the EIC signals were similar for all the mature SHPs (Table 3; Tables S14 and S15). It is important to note that absolute quantification of mature SHPs is difficult due to their high hydrophobicity and adhesive properties in LC-MS conditions developed for the analysis.

The RRNPP regulators that we identified in *S. thermophilus* also contained representatives of the PlcR family (TprA clusters) (11). As one such representative is present in *S. thermophilus* JIM8232, we used a concentrated supernatant of the strain to look for the presence of mature AIP PapR_Sthermo_1_. Employing LC-HR-MS/MS, we detected a peptide corresponding to the C-terminal of the precursor PapR_Sthermo_1_ (Fig. S13).

### Detection of RaS-RiPPs with cyclization motifs secreted by *S. thermophilus,* including a new enteropeptin

Using *in silico* screening, we identified five *shp*/*rgg* genes in *S. thermophilus* that are located in front of an operon involved in the production of five RaS-RiPPs: streptide (SHP/Rgg_gp_sali_6_), streptosactin (SHP/Rgg_Sthermo_13_), bicyclostreptin (SHP/Rgg_gp_sali_4_), enteropeptin (SHP/Rgg_gp_sali_5_), and ryptide (SHP/Rgg_gp_sali_7_) (Tables S2 and S5). We employed the strains JIM8232 and CIRM30 to detect the enteropeptin and the ryptide, respectively, for the first time ever in *S. thermophilus*. We used the strain JIM8232 to detect three RaS-RiPPs previously observed in *S. thermophilus* (streptide, streptosactin, and bicyclostreptin).

A homologous enteropeptin locus without *shp*/*rgg* genes is present in *Enterococcus cecorum*; it has been associated with three RaS-RiPP variants derived from the same precursor (enteropeptins A, B, and C) (39). These RaS-RiPPs are composed of seven to nine amino acids and feature a cyclization motif that links the sulfur of the Cys at position 5 and the alpha carbon of the Arg (converted into N-methyl-ornithine) at position 4; as such, these RaS-RiPPs belong to the sactipeptide family. Although the amino acid sequences of the precursor peptides differ between *S. thermophilus* and *E. cecorum,* several amino acids of the mature enteropeptin are conserved, including the amino acids R (Arg) and C (Cys) that are involved in the post-translational modification. We hypothesized that the same cyclization motif was present in our target peptides, and we searched for mature peptide forms in a concentrated supernatant (250×). We identified a similarly modified nine-amino-acid peptide via mass spectrometry, which we named enteropeptin D (Table 4; Fig. S14).

**TABLE 4:** Detection of RaS-RiPPs by LC-HRMS/MS in the supernatant of *S. thermophilus* strain JIM8232

Peptide	Mature sequence	Modification *m/z*	JIM8232^*a*^		
			RT^*b*^	EIC^*c*^ without concentration	EIC^*c*^ with concentration (250×)
Streptide	AKGDGWKVM	−2.01565	4.06	8.2E+6	5.5E+8
Bicyclostreptin	SWSKSHGH	−4.03130	1.88	7.5E+6	3.6E+6
Enteropeptin	LKGRCPPSV	−30.0218	7.52	N.D	1.0E+4
Streptosactin	NASCGPSHSCGGGR	−4.03130	3.45	N.D	8.1E+5

^*a*^ N.D, not detected.

^*b*^ RT, retention time (min).

^*c*^ Extracted ion chromatogram, area under the curve of the ion current signal.

We looked for the ryptide in strain CIRM30 but did not detect its presence, despite the fact that its encoding operon is complete in this strain. This finding fits with our observation that mature SHP_gp_sali_7_ was absent in the same strain.

We optimized a gradient method utilizing LC-HR-MS/MS in addition to exploiting the rapid growth of strain JIM8232 in adapted chemically defined media (CDMa) to determine the optimum production conditions of the three previously identified RaS-RiPPs (streptide, streptosactin, and bicyclostreptin) for rapid detection. Indeed, in the past, these three RaS-RiPPs had been detected using concentrated supernatants recovered after non-optimal strain growth (33, 34, 40). As a result, we were able to detect the streptide and the bicyclostreptin A directly from crude supernatants of this strain without the need for purification steps and rendered the detection process more straightforward for future experiments (see the paragraph below) (Table 4; Fig. S15 and S16). The streptosactin was also detected but only when using a concentrated supernatant (250×) (Fig. S17). Additionally, this experiment was conducted in a Δ*pptAB* background. As the *pptAB* gene encodes the exporter of the mature SHPs, its deletion should prevent the expression of the SHP/Rgg targets, including the genes involved in RaS-RiPP production (32). As expected, streptide and bicyclostreptin A were not detected in the crude supernatant of a JIM8232 Δ*pptAB* strain, confirming that they are regulated by an SHP/Rgg system.

In conclusion, all the RaS-RiPPs that are encoded by the genomes of JIM8232 are synthesized and secreted in chemically defined media (CDM). We also identified a new RaS-RiPP in *S. thermophilus*: enteropeptin D.

### Unlike RaS-RiPPs, mature SHPs accumulate temporarily in the extracellular medium during *S. thermophilus* growth

Research has yet to characterize mature SHP accumulation dynamics in the extracellular medium during growth, in contrast to research that has been done on RaS-RiPP (streptide, bicyclostreptin A, and streptosactin) dynamics (33, 34). Furthermore, the growth medium used in the latter studies was far from optimal for *S. thermophilus*.

**Fig 6:**
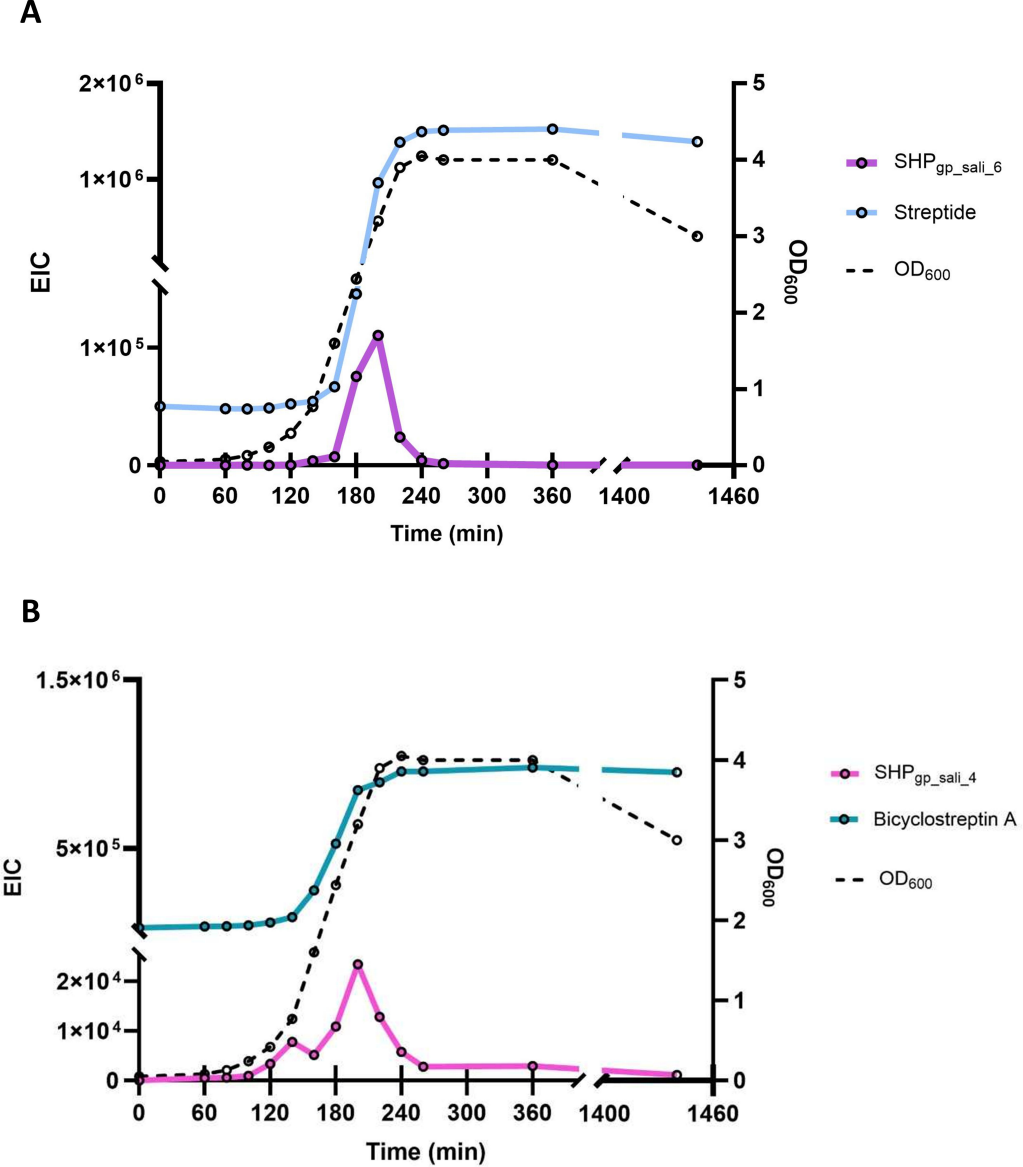
Production of the mature SHP_gp_sali_6_ and streptide (**A**) and mature SHP_gp_sali_4_ and bicyclostreptin (**B**) during growth of *S. thermophilus* strain JIM8232 in CDM. Bacterial growth (OD_600_) is represented in black dotted lines. The extracted ion chromatogram of the mature SHPs and RaS-RiPPs is represented by purple and cyan blue curves, respectively. Data shown are representative of two independent experiments.

To simultaneously monitor the production of both peptide types (RaS-RiPPs and SHPs) under optimal growth conditions in CDMa, we focused on streptide with SHP_gp_sali_6_ and bicyclostreptin A with SHP_gp_sali_4_ because these RaS-RiPPs can be detected without the use of concentrated supernatant. The latter is advantageous because concentrating the supernatant can cause variability in peptide yield. The quantities of the RaS-RiPPs and mature SHPs were estimated utilizing the area under the curve of their EIC signals. Levels of SHP_gp_sali_6_ became detectable after 150 min of growth, increased until 200 min of growth, and then started to decrease, before becoming undetectable after 260 min of growth (Fig. 6A). In sharp contrast, streptide levels were detectable at *T* = 0, indicating that the peptide was present in the preculture; levels increased until 240 min of growth and remained stable after 24 h. The production of SHP_gp_sali_4_ and bicyclostreptin A displayed a similar behavior to that of SHP_gp_sali_6_ and streptide, except that SHP_gp_sali_4_ was detected earlier during growth (Fig. 6B).

These data suggest that RaS-RiPPs are produced during the exponential growth phase and that they are then neither degraded in the supernatant nor reimported, probably because of cyclization. Mature SHPs also seem to be produced during the exponential growth phase but then disappear, reimported by the ABC transporter Ami to activate their cognate Rgg. To determine whether the other mature SHPs displayed a similar behavior, we monitored the levels of SHP_gp_sali_5_, SHP_Sthermo_6_, and SHP_Sthermo_13_ in the crude supernatants. In each case, mature SHP concentrations peaked in the middle of the exponential growth phase, then decreased between 180 and 200 min of growth, and rapidly disappeared at the beginning of the stationary phase (Fig. S18). The only notable difference in dynamics was that SHP_Sthermo_13_ occurred at detectable levels much earlier compared to the other mature SHPs.

**Fig 7:**
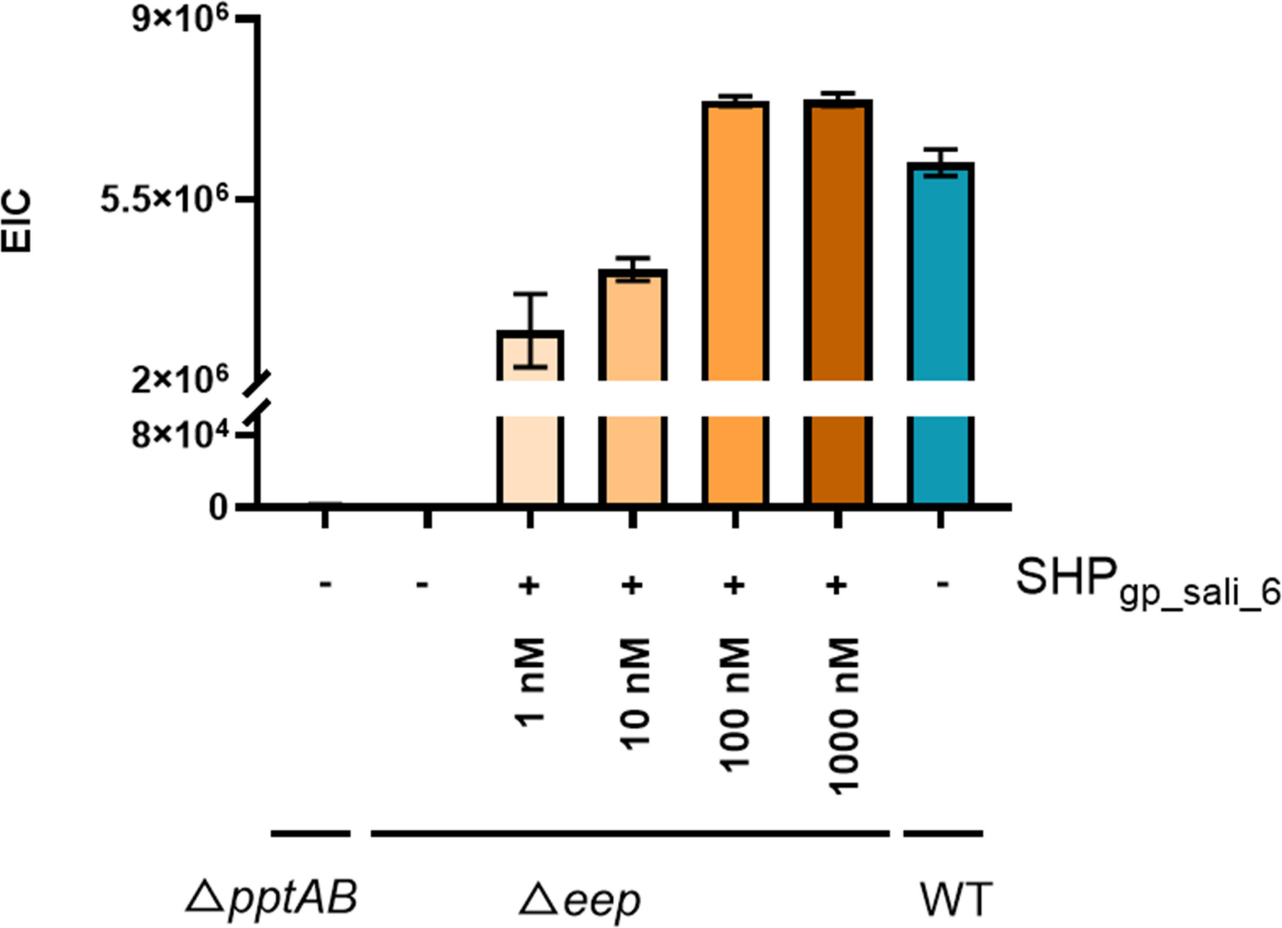
Production of streptide in strain TIL773 (Δ*eep*) after adding mature SHP_gp_sali_6_ at various concentrations in the CDM growth medium. Strains TIL773 and TIL1486 (Δ*pptAB*) grown without mature SHP_gp_sali_6_ are negative controls, and wild-type strain LMD-9 grown in the same condition is used as a positive control.

Finally, we assessed whether it was possible to increase the production of RaS-RiPPs by adding synthetic SHPs. We introduced SHP_gp_sali_6_ at various concentrations into the growth medium of strain TIL773 (Δ*eep*) and subsequently detected the presence of streptide. Eep is the protease involved in the maturation of SHP precursors. Similar to the *pptAB* deletion, deleting *eep* is expected to inhibit the expression of the SHP/Rgg targets, which include all genes involved in RaS-RiPP and SHP production. As shown in Fig. 7, we observed functional complementation of the Δ*eep* mutation with the addition of SHP_gp_sali_6_ at concentrations ranging from 1 to 1,000 nM. This effect was correlated with the SHP concentration in the medium. The maximum level of streptide production was achieved at a concentration of 100 nM, and this level was only slightly higher than that observed in the LMD-9 WT strain. As anticipated, no streptide was detected in the negative control strains TIL773 (Δ*eep*) and TIL1486 (Δ*pptAB*).

## DISCUSSION

We used genome mining to explore the repertoire of regulators belonging to the RRNPP superfamily in the salivarius group of streptococci. Our results revealed major differences in the RRNPP repertoires of *S. thermophilus* and *S. salivarius*. We found that the typical *S. thermophilus* genome encodes a large diversity of Rgg-family regulators but only a single ComR-family regulator, the ComS/ComR system that regulates competence. In contrast, we found that the typical *S. salivarius* genome encodes a ComS/ComR system accompanied by extra ComR-family regulators and a smaller number of Rgg-family regulators.

These differences are surprising, considering how closely related these two species are. In fact, *S. thermophilus* was classified as a subspecies of *S. salivarius* for several years, until it recently regained species status based on the results of different genomic studies (26, 27). *Streptococcus thermophilus* appears to have recently diverged from *S. salivarius* and is undergoing regressive evolution linked to niche specialization (28, 29). The plasticity of these species’ RRNPP repertoires (i.e., intraspecific differences among strains and the presence of transposase-encoding gene fragments) suggests that the RRNPP repertoire of a given strain may be the result of relatively rapid gene turnover. The two species share some Rgg clusters, suggesting the latter existed prior to the divergence of *S. thermophilus*. These shared clusters are more frequently found in *S. thermophilus* strains than in *S. salivarius* ones. In addition, other Rgg clusters might have been acquired by *S. thermophilus* from other streptococci present in farm environments, as these streptococci may have very similar loci (19). In parallel, the extra ComRs found in *S. salivarius* strains may have been lost as the species is undergoing regressive evolution.

We experimentally detected almost all of the most common mature SHPs across the different *S. thermophilus* strains, indicating that these systems have remained functional, which suggests they play an important role in milk or in farm environments. This conclusion was supported by the presence of enteropeptin D, in addition to the previously identified RaS-RiPPs. This result underscores that the four RaS-RiPP operons are potentially controlled by four SHP/Rgg systems capable of producing four different RaS-RiPPs. Our work is also the first to show that *S. thermophilus* synchronizes the secretion of these RaS-RiPPs into the extracellular medium. It is known that three RaS-RiPPs display a narrow spectrum of biological activity: the enteropeptin of *E. cecorum* (39) is bacteriostatic; the streptosactin likely has fratricidal effects (33); and the bicyclostreptin of *S. thermophilus* (34) has growth-regulated activity. It is worth noting that levels of streptide and bicyclostreptin A remained stable in the supernatant for 24 h. The cyclization motif of these two Ras-RiPPs likely provides increased resistance to enzymatic hydrolysis and prevents reimportation by the strain of origin. The synchronization in the production of RaS-RiPPs suggests a synergistic effect, as observed during the combined use of antibiotics in human health contexts (41). This hypothesis warrants experimental validation, such as comparing the antimicrobial properties of combinations of RaS-RiPPs against those of individual RaS-RiPPs added in culture media. We observed that the mature SHPs, which are non-modified peptides, disappeared during the second part of the exponential phase. This temporal profile likely results from the dynamics of peptide production and reimportation. Production predominates at the beginning of the exponential phase, while reimportation predominates at the end. This balance likely allows for the fine-tuning of the response to the mature SHP signals and limits interference that could result in extracellular mature SHPs with long half-lives.

In *S. thermophilus*, half of the Rgg clusters are located near the genes encoding ThiF enzymes or SAM radical enzymes. ThiF-modified peptides have also been observed to have antimicrobial properties (42–44). It seems probable that several Rgg-associated peptides modified by SAM radical enzymes or ThiF enzymes have antibacterial functions. This trend is less pronounced in *S. salivarius*. Interestingly, XIP-associated ComR appears to regulate the production of numerous bacteriocins in *S. salivarius* (45) but only a few bacteriocins in *S. thermophilus* (46). Moreover, to our knowledge, experimental research has only looked at two extra ComR regulators, and both are directly or indirectly associated with bacteriocin production (47). Although the AIPs of these ComR regulators have not been identified, genetic screening results have shown that synthetic peptides can control their activity. Therefore, *S. thermophilus* and *S. salivarius* appear to share a common strategy involving the production of antimicrobial peptides under the control of RRNPP regulators. However, for reasons that have yet to be elucidated, *S. thermophilus* uses Rgg regulators to control production, while *S. salivarius* uses ComR regulators to partially control production.

We projected the three SHP/Rgg groups that we had previously identified in Bacillota (Table S2; Fig. 5) (30) onto the Rgg repertoire of *S. thermophilus*. According to their relative positions in the phylogenetic tree, groups I and II diverged after the separation of group III. Group I contains three members that are specific to newly sequenced strains, in addition to two previously identified members. We detected putative mature SHPs for two of these members: one with a canonical sequence and one with an unusual Ala at the beginning of the mature form. Group II contains six SHP/Rgg members that had been identified in previous studies (14, 25, 30). Group III contains two AIP-associated RRNPP clusters: SHP/Rgg_Sthermo_6_ and MutS/MutR_Sthermo_5_. According to a recent work based on a broad collection of streptococcal QS, these clusters now belong to the MutRS cluster, specifically the MutRε for SHP/Rgg_Sthermo_6_ and MutRα subclusters for MutS/MutR_Sthermo_5_ (37). This new classification relies on multiple criteria: regulator homology, genomic orientation, peptide sequence, and promoter architecture. Interestingly, the amino acid sequence mature SHP_Sthermo_6_ (DIIIFPPFG) resembles that of the canonical group I mature SHP sequence (DIIIIVGG) and the MSP sequence (VLPFFLLPIL), the active mature form of the inactive precursor MutS. The X-ray crystal structure is also used as a criterion for defining a new RRNPP family, as demonstrated by ComRS (48). The structures of Rgg regulators from group I have been described (4, 49). It would be helpful to characterize the structures of one member of the MutR clusters α and ε and Rgg regulators (and their AIPs) from group II similarly, with a view to determining whether they have a specific mechanism of action. This will help determine if they can be considered as new families of the RRNPP superfamily or if they function as SHP/Rgg regulators of group I and can be considered as members of the SHP/Rgg subfamily. Taken together, these results underscore the difficulty of detecting AIPs based on similarities using validated sequences, even if coevolution between AIP sequences and regulators is observed. Therefore, we provide a larger list of putative peptide-encoding genes in Table S2 to facilitate the future discovery of AIPs with new sequence characteristics.

Finally, since we observed sequence similarities between the mature SHPs of *S. thermophilus*, we are currently studying the degree of recognition specificity between mature SHPs and Rgg regulators, paying particular attention to systems that may cross-activate.

## MATERIALS AND METHODS

### Detection of RRNPP genes with a profile HMM

Genomes and proteomes of all the bacterial isolates belonging to the salivarius group of streptococci were retrieved from NCBI RefSeq (files _genomic.gbff and _translated_cds.faa) in December 2023. In the RRNPP superfamily, the transcriptional regulators Rgg, PrgX, PlcR, and ComR families share a similar topology characterized by an N-terminal DNA-binding HTH domain and a C-terminal TPR domain with five TPR repeats, which makes it possible to build a generic HMM profile for their identification. To build this profile, we selected 33 baits (Table S1) with experimental data confirming that their activity is controlled by an AIP and/or the presence of a solved structure in the PDB database. This list included five putative RRNPP regulators (Rgg, ElrA, GadR, LasX, and lmo0325), belonging to the Rgg family in the broad sense, which have been the subject of experimental investigation but for which there is no evidence of activation by an AIP. The 33 protein sequences were submitted to the PROMALS3D web server (http://prodata.swmed.edu/promals3d/, accessed in November 2021 and used with default parameters) to construct an alignment using information from sequence database searches, secondary structure prediction, and available 3D structures (50). This 1D/3D alignment was confirmed using PDBeFold (https://www.ebi.ac.uk/msd-srv/ssm/) and visually inspected with respect to the superposition on the five TPR repeat domains of the solved structures, using PyMOL (The PyMOL Molecular Graphics System, version 2.1, Schrödinger, LLC) (51).

The HMM profile was built from the protein sequence alignment of 33 baits using hmmbuild (HMMER version 3.3.2 [32]), with default parameters. The length of this profile is 286 amino acids. RRNPP regulators were then identified by searching this HMM profile in the database of 527 proteomes with hmmsearch (HMMER version 3.3.2). A total of 2,900 statistically significant RRNPP hits (*E*-value ≤ 1e-5), with alignment to the entire or nearly entire HMM profile (starting from position ≤ 10 and coverage ≥ 90%), were identified. Detection with hmmsearch also produced a multiple sequence alignment of the hits. Columns of this alignment were filtered to retain the 267 with high consensus posterior probability (“PP_cons” annotation “9” and “*”). RRNPP similarity clusters (Table S2; column RRNPP cluster) were defined as connected components after applying a cutoff of 90% identity calculated on this alignment.

### Prediction of small coding sequences and identification of AIPs

Short coding sequences (CDS ≤ 60 aa) were detected in genome sequences, following the same principle as in references 14 and 30, with two minor modifications to improve the consistency of predictions between isolates. First, a unique HMM for CDS detection was applied to the 527 isolates. This model was estimated from sequence composition properties on the genome sequences of five representative isolates covering a maximum diversity of RRNPP regulators (NCBI RefSeq records GCF_950102085.1, GCF_009717065.1, GCF_027857875.1, GCF_023109245.1, and GCF_003458505.1). Second, for each genome, predictions were made on both DNA strands using exactly the same CDS model (this was achieved by considering the positive-strand prediction on the genome sequence and its reverse complement). Predictions of length <7 aa were discarded. As in reference 30, predictions with a confidence probability ≥ 0.01 were considered putative short CDSs. Table S2 lists all putative short CDSs within 300 bp on both sides of the RRNPP hits.

In the neighborhood of the RRNPP genes, the short CDSs with probable AIP function were manually selected on the basis of similarity to the sequences of SHP, ComS, PapR, and LCP peptides already published for some strains (11, 13, 24, 30).

### Classification, phylogeny, and genomic context of RRNPP genes

RRNPP families and subfamilies were automatically assigned to the hits by transferring this information from their closest blastp (52) match among the 33 baits (Table S1). Each cluster of Table S2 was annotated with the name of its RRNPP subfamily and, if relevant, the name of the AIP, followed by a unique identifier. If the regulator was specific to one species, the identifier referred to this species, e.g., SHP/Rgg_Sthermo10_, and if the regulator was found in at least two species, the identifier referred to the group salivarius, e.g., Rgg-like_gp_Sali2_. The same identifier was used for the annotation of the AIP, e.g., SHP_Sthermo10_.

Phylogenetic trees were reconstructed for the RRNPP hits using FastTree version 2.1.10 (53) based on the HMMER 267-column alignment with high consensus posterior probability. Branch support was calculated by resampling the site likelihoods 1,000 times and using the Shimodaira-Hasegawa test. Graphical representations of these trees were generated using the ETE3 Programmable Tree Drawing Engine (54) with midpoint rooting.

NCBI annotated CDSs in a genomic neighborhood of 2 kbp, on both sides of the RRNPP genes, were compared in an all-against-all blastp version 2.12 comparison (*E*-value ≤ 1e-3). Clusters were defined as connected components using a cutoff of alignment coverage ≥ 70% of both sequences in pairwise comparisons (Table S2).

### Bacterial strains and growth conditions

The *S. thermophilus* strains used in this study were LMD-9 (55), TIL773 (LMD-9 Δ*eep*) (30), TIL1486 (LMD-9 Δ*pptAB*) (32), JIM8232 (56), CNRZ1066 (29), N4L (57), and CIRM30 (direct submission, NZ_LR822012). They were grown at 37°C without shaking, under atmospheric air in chemically defined media (CDM) (58), except strain JIM8232, which was grown in an adapted CDM (CDMa) (59) to avoid flocculation. Optical density at 600 nm (OD_600_) was measured using a Biochrom Libra S11 spectrophotometer.

### Sample preparation for LC-HR-MS/MS

Five milliliters of culture of *S. thermophilus* strains were grown in 15 mL Falcon tubes until they reached an OD_600_ of 0.8 (JIM8232) or 1.1 (±0.1) (CIRM30, LMD-9, N4L, and CNRZ1066), values corresponding to the mid-exponential growth phase. Supernatants (500 µL) were recovered from 1 mL of culture via centrifugation, and formic acid was added at a final concentration of 1% to inactivate any potential degradative enzymes that had been secreted. The acidified supernatants were flash-frozen in liquid nitrogen and stored at −70°C until the LC-HR-MS/MS could be performed. To characterize SHP/RaS-RiPP dynamics, samples (1 mL) were collected at *T* = 0, every 20 min between *T* = 60 min and *T* = 6 h, and at *T* = 24 h from 40 mL of culture grown in 50 mL Falcon tubes.

SHP_gp_sali_3_ starts with a cysteine residue. To detect this SHP using LC-HR-MS/MS, we alkylated its thiol functional group using iodoacetamide (IAA) under reductive conditions induced with dithiothreitol (DTT). We incubated 1 mL of supernatant or 1 mL of synthetic peptide (100 nM in DMSO) in DTT (10 mM) for 45 min at 56°C; IAA (55 mM) was then added to the sample, which was placed in the dark at room temperature for 45 min. The samples were then purified using a Strata-X cartridge (Phenomenex) containing 1 mL of 80% acetonitrile; they were subsequently speed-vacuum dried and resuspended in a loading buffer (supplemental material and methods) before being injected into the mass spectrometer.

To detect the presence of enteropeptin D and streptosactin, a concentrated supernatant was prepared from 50 mL of cells grown in a 50 mL Falcon tube. We concentrated the supernatant on Sep-Pak C18 Plus 330 mg (Waters), which was washed with 10 mL of 0.1% formic acid and eluted with 2 mL of 80% acetonitrile. After speed-vacuum drying, the samples were resuspended in 200 µL of loading buffer and then injected (2 µL) into the mass spectrometer.

Two biological replicates were prepared for all conditions tested, except for the detection of enteropeptin and streptosactin, for which only one supernatant was processed.

### Synthesis of SHP peptides

The synthetic peptides were purchased with a purity of 90% or higher from the following companies: GenScript for SHP_gp_sali_5_ (ESIIVIAVG); GeneCust for SHP_Sthermo_6_ (DIIIFPPFG) and SHP_Sthermo_13_ (EGIIVIGVG); Covalab for SHP_gp_sali_6_ (EGIIVIVVG) and SHP_gp_sali_7_ (EGIIVILVG); Proteogenix for SHP_gp_sali_4_ (EIIIIIAL), SHP_gp_sali_3_ (CIYTIVGGV), and SHP_Sthermo_12_ (DIIIIVGG). They were resuspended as 1 mM stock in dimethyl sulfoxide.

### LC-HR-MS/MS analysis

The presence of synthetic and natural SHPs was detected using liquid chromatography-high resolution tandem mass spectrometry using two device configurations. Configuration 1 is a nanoRSLC chromatographic system (Thermo Fisher Scientific) coupled via a nanoelectrospray (nanoESI) ion source with an Orbitrap Fusion Lumos Tribrid mass spectrometer (Thermo Fisher Scientific), and configuration 2 is a Vanquish Flex LC system coupled via an electrospray (ESI) ion source to a Q Exactive Focus mass instrument (Thermo Fisher Scientific).

The most sensitive configuration (configuration 1) was used to detect all the natural SHPs using MS/MS. SHP detection was validated via manual annotation of the MS/MS spectra. To confirm that the sequences of the natural SHPs had been properly identified, a synthetic version of each SHP was resuspended in the CDM medium at a concentration of 125 nM and then injected into the mass spectrometer under the same conditions as the supernatant; the retention times and mass spectra of the natural and artificial SHPs were compared. Several SHPs were found in the supernatant of the different strains. However, for illustrative purposes, we have only provided one representative MS/MS spectrum for a natural SHP accompanied by the MS/MS spectrum for the corresponding synthetic SHP.

A standard LC-ESI MS configuration (configuration 2) was used to identify the RaS-RiPPs based on MS/MS spectra from the literature (33, 34, 39). The enteropeptin in *S. thermophilus* is not identical to the enteropeptin in *Enterococcus cecorum*, but the amino acids involved in the cyclization motif are conserved; the differences were accounted for during the comparison. Configuration 2 was also used in the single ion monitoring mode of acquisition during the growth dynamics experiments, where the run of acquisition was optimized (15 min) for each time point.

The LC and MS methodologies are described in detail in the supplemental material and methods.

## SUPPLEMENTAL MATERIAL

Supplemental tables jb.00231-25-s0001.docx Tables S3 to S15.

Supplemental figures jb.00231-25-s0002.ppt Figures S1 to S18.

Supplemental material and methods jb.00231-25-s0003.docx Complement to the LC-HR-MS/MS analysis.

Table S1 jb.00231-25-s0004.xlsx List of the baits used to construct the HMM profile for the detection of RRNPP regulators.

Table S2 jb.00231-25-s0005.xlsx Classification of RRNPP regulators of streptococci of the salivarius group.

ASM does not own the copyrights to Supplemental Material that may be linked to, or accessed through, an article. The authors have granted ASM a non-exclusive, world-wide license to publish the Supplemental Material files. Please contact the corresponding author directly for reuse.
