# Supplementary material for: RRNPP quorum-sensing repertoires in the salivarius group genomes: overrepresentation and synchronous activation of SHP/Rgg systems in Streptococcus thermophilus
Source: J Bacteriol. 2025 Aug 26;207(9):e00231-25. doi: 10.1128/jb.00231-25 (PMC12445099; doi:10.1128/jb.00231-25)
Supplement: Supplemental tables — Tables S3 to S15. [file jb.00231-25-s0001.docx]

**TABLE S3** RRNPP clusters in *Streptococcus salivarius*

| **RRNPP family** | **RRNPP**  **cluster**  **identifier^1^** | **Putative AIP^2,3,4^** | **Strain/**  **Isolate^5^** | **Blastp best bait identifier** | **% identity**  **(coverage)** |
| --- | --- | --- | --- | --- | --- |
| ComR | ComR-like_Ssali_1_ (66) | N.D. | HS0302 | bait17_ComRSs | 33(98) |
| ComR | ComS/ComR_Ssali_2_ (14) | MKNLKKFLVLLIAVAPFFINYY (14) | 1003_SOLI | bait18_ComRSv | 90(100) |
| ComR | ComS/ComR_gp_sali_1_ (190) | MKKLKLFTLFSLLITILPYFAGCL (37) | BIOML-A29 | bait16_ComRSt | 95(100) |
| ComR | ComR_Ssali_3_ (78) | N.D. | K12 | bait18_ComRSv | 41(97) |
| ComR | ComR_Ssali_4_ (1) | N.D. | UBSS01 | bait18_ComRSv | 43(92) |
| ComR | ComR_Ssali_5_ (23) | N.D. | B14011936 | bait18_ComRSv | 41(97) |
| ComR | ComR_Ssali_6_ (26) | N.D. | JIM8777  BIOML-A29 | bait18_ComRSv  bait16_ComRSt | 42(96)  40(95) |
| ComR | ComR_Ssali_7_ (1) | N.D. | PA11332C3 | bait16_ComRSt | 41(97) |
| ComR | ComR_Ssali_8_ (27) | N.D. | AF11-9H | bait18_ComRSv | 42(97) |
| ComR | ComR_Ssali_9_ (2) | N.D. | K12 | bait16_ComRSt | 42(95) |
| ComR | ComR_Ssali_10_ (16) | N.D. | CF7-5 | bait16_ComRSt | 42(95) |
| ComR | ComR_Ssali_11_ (60) | N.D. | ATCC 27945  UBSS01 | bait16_ComRSv  bait18_ComRSt | 42(94)  41(97) |
| ComR | ComR_Ssali_12_ (5) | N.D. | JIM8777 | bait16_ComRSt | 42(95) |
| ComR | ComR_Ssali_13_ (1) | N.D. | AM03-15X | bait18_ComRSv | 41(97) |
| PlcR | PapR/TprA-like_Ssali_1_ (11) | MKKYKKIASLSILLLLLFSVFNLNTINHENHLFNVVDPGAANGVSKYY (2)  MKKYKKIASLSILLLLLFSVFNLNTINHENHLFNVIDPGAANWVSKYY (2) | VA08-2AN  AM113-115 | bait28_TprA  bait28_TprA | 38(97)  38(97) |
| PlcR | PapR/TprA_Ssali_2_ (1) | MKKFITIIAVVFICIAIIGLGANFDRQLSITNQHVKSDRIDPGPANLF (1) | AF24-6AC | bait28_TprA | 42(97) |
| PlcR | PapR/TprA-like_gp_sali_1_ (37) | MKKRLKRLTAICLLSFLLISFGIGTDSGAKTSGRIDPGPAHSMVS (4)  MKKQLKKLTAICLLSFLLISFGIGTDSGVKTSGRIDPGPAHSIIS (1) | MGYG-HGUT-00113  84-12 S20 | bait28_TprA  bait28_TprA | 39(98)  40(98) |
| Rgg | Rgg-like_gp_sali_1_ (4) | N.D. | 37-08 S12  726_SSAL | bait5_SHP/RovS bait27_Rgg | 28 (66)  26(95) |
| Rgg | Rgg-like_Ssali_1_ (144) | N.D. | ATCC 27945 | bait5_SHP/RovS | 21(95) |
| Rgg | LCP/RopB_Ssali_2_ (2) | MWLILLFL (2) | K12 | bait10_LCP/RopB | 52 (96) |
| Rgg | Rgg-like_Ssali_3_ (8) | N.D. | FDAARGOS_1045  AMBR158 | bait4_SHP/RggSt0182  bait14_SHP/RggSpn1518 | 28(89)  26(62) |
| Rgg | Rgg-like_Ssali_4_ (5) | N.D. | AM110-184  AM113-115 | bait4_SHP/RggSt0182  bait14_SHP/RggSpn1518 | 29(68)  27(62) |
| Rgg | Rgg-like_Ssali_5_ (3) | N.D. | ICDC2 | bait27_Rgg | 36(97) |
| Rgg | Rgg_Ssali_6_ (1) | N.D. | MIT 14-1770-C1 | bait27_Rgg | 46(96) |
| Rgg | SHP/Rgg_Ssali_7_ (3) | MKKQFNLCVVLFELIIIISPSLIFL (1) | AF23-9AC | Bait2_SHP/RggSt1358 | 55(97) |
| Rgg | SHP/Rgg_gp_sali_4_ (2) | MNISIKRFLMIILEIIIIIAL (2) | DB-B5 | Bait3_SHP/RggSmu1509 | 47(98) |
| Rgg | SHP/Rgg_gp_sali_5_ (3) | MNKESFFAILLLIFESIIVIAVG (1) | MIT 14-1770-C1 | Bait2_SHP/RggSt1358 | 63(99) |
| Rgg | SHP/Rgg_gp_sali_7_ (116) | MKKQKLLLLVVLVCEGIIVILVG (1)  MKKQKLLLLVVSVCEGIIVILVG (2) | ICDC2  39-01 S14 | Bait2_SHP/RggSt1358  Bait2_SHP/RggSt1358 | 80(98)  80(98) |
| Rgg | SHP/Rgg_Ssali_8_ (1) | MKKQQLLSFLLLLFEGILVIVVG (1) | GCF_902850305.1 | Bait2_SHP/RggSt1358 | 87(99) |
| Rgg | SHP/Rgg_gp_sali_6_ (8) | MKKQNLLTLLLVVFEGIIVIVVG (8) | ATCC 25975 | Bait2_SHP/RggSt1358 | 100(100) |
| Rgg | SHP/Rgg_Ssali_9_ (2) | MMKNISKLIPILILFIDIIIIAGG (2) | D40t1_170626_E6 | Bait11_SHP/RggSpn0939 | 81(100) |
| Rgg | SHP/Rgg_Ssali_10_ (4) | MKKLLKIFPILFLFLDIIIIVGG (4) | AMBR024 | Bait13_SHP/RggSmi0094 | 74(99) |
| Rgg | SHP/Rgg_Ssali_11_ (2) | MKKISKLIPILLFFLDIIIIVGG (2) | M18 | Bait11_SHP/RggSpn0939 | 72(100) |
| Rgg | SHP/Rgg_Ssali_12_ (2) | MEKILKILSILLIILDIIIIVG (2) | D40t1_170626_E6 | Bait11_SHP/RggSpn0939 | 75(99) |
| Rgg | SHP/Rgg_Ssali_13_ (1) | MKKISKLLSILILVTDIIIIIGS (1) | 1001216st2_A8_1001216I_160404 | Bait11_SHP/RggSpn0939 | 83(98) |
| Rgg | Rgg-like_gp_sali_8_ (11) | N.D. | GCF_903557885.1  AMBR055 | bait27_Rgg  bait8_SHP/Rgg2Sd | 32(91)  34(96) |
| Rgg | Rgg-like_gp_sali_2_ (193) | N.D. | GCF_903910465.1  HSISS4 | bait27_Rgg  bait8_SHP/Rgg2Sd | 32(91)  33(96) |

^1^The hit number is in brackets.

^2^In bold, the sequence of a mature form of the AIP as observed experimentally.

^3^N.D. Not detected (no peptide detected or no obvious AIP signature for a detected peptide).

^4^Mutations in comparison to the most common form are higlighted in grey.

^5^One strain and its AIP (the first in the list in Table S2) was selected for each RRNPP cluster.

**TABLE S4** RRNPP clusters in *Streptococcus vestibularis*

| **RRNPP family** | **RRNPP**  **cluster**  **identifier^1^** | **Putative AIP^2,3^** | **Strain/**  **Isolate^4^** | **Blastp best bait identifier** | **% identity**  **(coverage)** |
| --- | --- | --- | --- | --- | --- |
| ComR | ComS/ComR_gp_sali_1_ (3) | MKTLKIFVLFSLL**IAILPYFAGCL** (3) | Bg39 | bait16_ComRSt | 95(100) |
| ComR | ComS/ComR_Svesti_1_ (28) | MKNLKKFLVLLIAAVPFFMIYY (21) | O4-4 | bait18_ComRSv | 98 (100) |
| Rgg | SHP/Rgg_gp_sali_5_ (2) | MNKESFLAILLLIFEGIIVIAVG (2) | S06 | Bait2_SHP/RggSt1358 | 62 (99) |
| Rgg | SHP/Rgg_Svesti_1_ (1) | MKNNFLSKFLIVLANILIIITM (1) | S06 | bait27_Rgg | 46 (96) |
| Rgg | SHP/Rgg_gp_sali _3_ (1) | MKLLKIIVLLTCIYIIVGGV (1) | DP4_2A | bait4_SHP/RggSt0182 | 99 (100) |
| Rgg | Rgg-like_gp_sali_8_ (6) | N.D. | MGYG-HGUT-02302 | bait27_Rgg | 31 (91) |
| Rgg | Rgg-like_gp_sali_2_ (22) | N.D. | DP3_2B_2  ERR9609794_bin.7_MetaWRAP_v1.3_MAG | bait8_SHP/Rgg2Sd  bait27_Rgg | 33 (96)  32 (91) |

^1^The hit number is in brackets.

^2^In bold, the sequence of a mature form of the AIP as observed experimentally.

^3^N.D. Not detected (no peptide detected or no obvious AIP signature for a detected peptide).

^4^One strain and its AIP (the first in the list in Table S2) was selected for each RRNPP cluster.

**TABLE S5** Functional annotations found in the vicinity of the *rgg* genes in *Streptococcus thermophilus*

| **RRNPP family** | **RRNPP**  **cluster**  **with identifier^1^** | **Functional annotations^2^** | **Sens^3^** |
| --- | --- | --- | --- |
| Rgg | Rgg-like_gp_sali_1_ (36) | **Radical SAM protein, Transporter (36)** | **⬅⬅⮕** |
| Rgg | Rgg-like_Sthermo_1_ (89) | **Radical SAM protein, Transporter (76)** | **⬅⬅⮕** |
| Rgg | LCP/RopB_Sthermo_2_ (13) | **Radical SAM protein, Transporter (5)** | **⬅⬅⬅⮕** |
| Rgg | Rgg-like_gp_sali_2_ (3) | **Transporter (3)** | **⮕⮕** |
| Rgg | Rgg-like_Sthermo_3_ (174) | **ThiF (3)** | **⬅⮕** |
| Rgg | Rgg-like_Sthermo_4_ (3) | **ThiF, Transporter (3)** | **⬅⬅⮕⬅** |
| Rgg | MutS/MutR_Sthermo_5_ (2) | **Bacteriocin , Transporter (2)** | **⮕⬅⮕⮕⮕⮕** |
| Rgg | SHP/Rgg_Sthermo_6_ (157) | **ThiF (150), Transporter (8)** | **⮕⬅⮕⮕** |
| Rgg | Rgg-like_Sthermo_7_ (8) | **Transporter, Transporter (8)** | **⬅⬅⬅⮕** |
| Rgg | Rgg-like_Sthermo_8_ (3) |  |  |
| Rgg | SHP/Rgg-like_Sthermo_9_ (2) |  | **⬅⮕** |
| Rgg | SHP/Rgg_Sthermo_10_ (1) |  | **⬅⮕** |
| Rgg | Rgg_Sthermo_11_ (5) | **Transporter, Transporter (5)** | **⬅⬅⬅⬅⮕** |
| Rgg | SHP/Rgg_Sthermo_12_ (46) |  | **⬅⮕** |
| Rgg | SHP/Rgg_gp_sali _3_ (109) |  | **⬅⮕** |
| Rgg | SHP/Rgg_gp_sali_4_ (27) | **Radical SAM protein, Radical SAM protein (27)** | **⬅⮕⮕⮕⮕** |
| Rgg | SHP/Rgg_Sthermo_13_ (35) | **Radical SAM protein, Transporter (35)** | **⬅⮕⮕⮕⮕** |
| Rgg | SHP/Rgg_gp_sali_5_ (262) | **Radical SAM protein (261)** | **⬅⮕⮕⮕** |
| Rgg | SHP/Rgg_gp_sali_6_ (187) | **Radical SAM protein, Transporter (185)** | **⬅⮕⮕⮕⮕** |
| Rgg | SHP/Rgg_Sthermo_14_ (14) | **Radical SAM protein, Transporter (14)** | **⬅⮕⮕⮕⮕** |
| Rgg | SHP/Rgg_gp_sali_7_ (265) | **Transporter (265)** | **⬅⮕⮕** |

^1^The hit number is in brackets.

^2^Transporters include ABC and MFS transporters. The hit number is in brackets.

^3^The hypothetical SHP, Rgg Regulators, Radical SAM protein, ThiF Enzyme, Transporter, bacteriocin, hypothetical protein are represented by black, red, purple, orange, blue,green and grey arrows respectively.

**TABLE S6** Functional annotations found in the vicinity of the *rgg* genes in *Streptococcus salivarius*

| **RRNPP family** | **RRNPP**  **cluster**  **with identifier^1^** | **Functional annotations ^2^** | **Genomic context^3^** |
| --- | --- | --- | --- |
| Rgg | Rgg-like_gp_sali_1_ (4) | **Radical SAM protein(2) Transporter (4)** | **⬅⬅⮕** |
| Rgg | Rgg-like_Ssali_1_ (144) | **Radical SAM protein (1), Transporter (143)** | **⬅⬅⮕** |
| Rgg | LCP/RopB_Ssali_2_ (2) |  | **⬅⮕** |
| Rgg | Rgg-like_Ssali_3_ (8) |  | **⮕** |
| Rgg | Rgg-like_Ssali_4_ (5) |  | **⮕** |
| Rgg | Rgg-like_Ssali_5_ (3) | **Transporter, Transporter (3)** | **⬅⬅⮕** |
| Rgg | Rgg_Ssali_6_ (1) | **Transporter, Transporter (1)** | **⮕⮕⮕⮕** |
| Rgg | SHP/Rgg_Ssali_7_ (3) |  | **⬅⮕** |
| Rgg | SHP/Rgg_gp_sali_4_ (2) | **Radical SAM protein, Radical SAM protein (2)** | **⬅⮕⮕⮕** |
| Rgg | SHP/Rgg_gp_sali_5_ (3) | **Radical SAM protein (2)** | **⬅⮕⮕⮕** |
| Rgg | SHP/Rgg_gp_sali_7_ (116) | **Transporter (116)** | **⮕⮕** |
| Rgg | SHP/Rgg_Ssali_8_ (1) | **Radical SAM protein (1)** | **⬅⮕⮕** |
| Rgg | SHP/Rgg_gp_sali_6_ (8) | **Radical SAM protein (7), Transporter (8)** | **⬅⮕⮕⮕** |
| Rgg | SHP/Rgg_Ssali_9_ (2) |  | **⬅⮕** |
| Rgg | SHP/Rgg_Ssali_10_ (4) |  | **⬅⮕** |
| Rgg | SHP/Rgg_Ssali_11_ (2) |  | **⬅⮕** |
| Rgg | SHP/Rgg_Ssali_12_ (2) |  | **⬅⮕** |
| Rgg | SHP/Rgg_Ssali_13_ (1) |  | **⬅⮕** |
| Rgg | Rgg-like_gp_sali_8_ (11) |  | **⮕** |
| Rgg | Rgg-like_gp_sali_2_ (193) | **Transporter (192)** | **⮕⮕** |

^1^The hit number is in brackets.

^2^Transporters include ABC and MFS transporters. The hit number is in brackets.

^3^The hypothetical SHP, Rgg Regulators, Radical SAM protein, ThiF Enzyme, Transporter, bacteriocin, are represented by black, red, purple, orange, blue and green arrows respectively.

**TABLE S7.** LC-HRMS/MS data for *S. thermophilus* SHP_Sthermo_6_

| **Sequence** | **Ion** | **Calculated m/z** | **Observed m/z**  **Natural** | **Observed m/z**  **Synthetic** |
| --- | --- | --- | --- | --- |
| **DI** | **b_2_^+1^** | 229.11833 | 229.11809 | 229.11811 |
| **DII** | **b_3_^+1^** | 342.20239 | 342.20170 | 342.20197 |
| **DIII** | **b_4_^+1^** | 455.28646 | 455.28600 | 455.28636 |
| **FPPFG** | **y_5_^+1^** | 564.28170 | 564.28065 | 564.28158 |
| **PPFG** | **y_4_^+1^** | 417.21329 | 417.21340 | 417.21281 |
| **PFG** | **y_3_^+1^** | 320.16053 | 320.16049 | 320.16011 |
| **DIIIFPPFG** | [M+H]^+^ | 1018.56084 | 1018.55794 | 1018.55770 |

**TABLE S8.** LC-HRMS/MS data for *S. thermophilus* SHP_gp_sali_6_

| **Sequence** | **Ion** | **Calculated m/z** | **Observed m/z**  **Natural** | **Observed m/z**  **Synthetic** |
| --- | --- | --- | --- | --- |
| **EGI** | **b_3_^+1^** | 300.15544 | 300.15504 | 300.15477 |
| **EGII** | **b_4_^+1^** | 413.23951 | 413.23925 | 413.23866 |
| **EGIIV** | **b_5_^+1^** | 512.30792 | 512.30861 | 512.30680 |
| **EGIIVI** | **b_6_^+1^** | 625.39198 | 625.39229 | 625.39047 |
| **EGIIVIV** | **b_7_^+1^** | 724.46040 | 724.46136 | 724.45900 |
| **EGIIVIVVG** | [M+H]^+^ | 898.56084 | 898.56003 | 898.57355 |

**TABLE S9.** LC-HRMS/MS data for *S. thermophilus* SHP_Sthermo_13_

| **Sequence** | **Ion** | **Calculated m/z** | **Observed m/z**  **Natural** | **Observed m/z**  **Synthetic** |
| --- | --- | --- | --- | --- |
| **EGI** | **b_3_^+1^** | 300.15544 | 300.15514 | 300.15480 |
| **EGII** | **b_4_^+1^** | 413.23951 | 413.23988 | 413.23908 |
| **EGIIV** | **b_5_^+1^** | 512.30792 | 512.30802 | 512.30810 |
| **EGIIVI** | **b_6_^+1^** | 625.39198 | 625.39294 | 625.39125 |
| **EGIIVIG** | **b_7_^+1^** | 682.41345 | 682.41424 | 682.41230 |
| **EGIIVIGV** | **b_8_^+1^** | 781.48186 | 781.48296 | 781.48085 |
| **EGIIVIGVG** | **b_9_^+1^** | 838.50332 | 838.50429 | 838.50248 |
| **EGIIVIGVG** | [M+H]^+^ | 856.51389 | 856.51392 | 856.51350 |

**TABLE S10.** LC-HRMS/MS data for *S. thermophilus* SHP_gp_sali_5_

| **Sequence** | **Ion** | **Calculated m/z** | **Observed m/z**  **Natural** | **Observed m/z**  **Synthetic** |
| --- | --- | --- | --- | --- |
| **ESI** | **b_3_^+1^** | 330.16601 | 330.16635 | 330.16610 |
| **ESII** | **b_4_^+1^** | 443.25007 | 443.25105 | 443.25044 |
| **ESIIV** | **b_5_^+1^** | 542.31848 | 542.32136 | 542.31886 |
| **ESIIVI** | **b_6_^+1^** | 655.40255 | 655.40653 | 655.40295 |
| **ESIIVIA** | **b_7_^+1^** | 726.43966 | 726.43932 | 726.44074 |
| **ESIIVIAVG** | [M+H]^+^ | 900.54010 | 900.53766 | 900.53859 |

**TABLE S11.** LC-HRMS/MS data for *S. thermophilus* SHP_gp_sali_4_

| **Sequence** | **Ion** | **Calculated m/z** | **Observed m/z**  **Natural** | **Observed m/z**  **Synthetic** |
| --- | --- | --- | --- | --- |
| **EII** | **b_3_^+1^** | 356.21804 | N.D | 356.21705 |
| **EIII** | **b_4_^+1^** | 469.30211 | 469.30271 | 469.30119 |
| **EIIII** | **b_5_^+1^** | 582.38617 | 582.38828 | 582.38491 |
| **EIIIII** | **b_6_^+1^** | 695.47023 | 695.47283 | 695.46843 |
| **EIIIIIA** | **b_7_^+1^** | 766.50735 | 766.50950 | 766.50597 |
| **EIIIIIAL** | [M+H]^+^ | 897.60197 | 897.60259 | 897.60014 |

**TABLE S12.** LC-HRMS/MS for *S. thermophilus* SHP_Sthermo_12_

| **Sequence** | **Ion** | **Calculated m/z** | **Observed m/z**  **Natural** | **Observed m/z**  **Synthetic** |
| --- | --- | --- | --- | --- |
| **DII** | **b_3_^+1^** | 342.20239 | 342.20281 | 342.20187 |
| **DIII** | **b_4_^+1^** | 455.28646 | 455.28819 | 455.28592 |
| **DIIII** | **b_5_^+1^** | 568.37052 | 568.37233 | 568.37023 |
| **DIIIIV** | **b_6_^+1^** | 667.43893 | 667.44105 | 667.43851 |
| **DIIIIVG** | **b_7_^+1^** | 724.46040 | 724.46220 | 724.45993 |
| **DIIIIVGG** | [M+H]^+^ | 799.49242 | 799.49106 | 799.49108 |

**TABLE S13.** LC-HRMS/MS data for *S. thermophilus* SHP_gp_sali_3_

| **Sequence** | **Ion** | **Calculated m/z** | **Observed m/z**  **Natural** | **Observed m/z**  **Synthetic** |
| --- | --- | --- | --- | --- |
| **CI** | **b_2_^+1^** | 274.12207 | N.D | 274.12283 |
| **CIY** | **b_3_^+1^** | 437.1854 | 437.18506 | 437.18704 |
| **CIYT** | **b_4_^+1^** | 538.23308 | 538.23380 | 538.23541 |
| **CIYTI** | **b_5_^+1^** | 651.31714 | 651.31819 | 651.32016 |
| **CIYTIV** | **b_6_^+1^** | 750.38555 | 750.38696 | 750.38896 |
| **CIYTIVG** | **b_7_^+1^** | 807.40702 | 807.40782 | 807.41087 |
| **CIYTIVGG** | **b_8_^+1^** | 864.42848 | 864.42919 | 864.43247 |
| **CIYTIVGG** | **b_9_^+1^** | 963.49689 | N.D | 963.50082 |
| **CIYTIVGGV** | [M+H]^+^ | 981.50746 | 981.50562 | 981.50578 |

**TABLE S14 :** Detection of natural SHPs by HRLC-MS/MS in the raw supernatant of *S. thermophilus* strains LMD-9 and N4L

| **Peptide** | **Mature sequence** | **RT^1^ of synthetic standard** | **LMD-9^2,3^** | | **N4L^2,3^** | |
| --- | --- | --- | --- | --- | --- | --- |
|  |  |  | RT^1^ | EIC^4^ | RT^1^ | EIC^4^ |
| SHP_gp_sali_5_ | **ESIIVIAVG** | 25.3 | 25.3 | 2.16E+05 | 25.3 | 1.64E+06 |
| SHP_Sthermo_6_ | **DIIIFPPFG** | 29.8 | 29.8 | 2.65E+06 | N.D | N.D |
| SHP_gp_sali_6_ | **EGIIVIVVG** | 26.2 | 26.1 | 5.38E+06 | 26.2 | 5.02E+06 |
| SHP_gp_sali_7_ | **EGIIVILVG** | 26.0 | N.D | N.D | N.D | N.D |
| SHP_Sthermo_13_ | **EGIIVIGVG** | 25.2 | 25.1 | 1.26E+06 | 25.1 | 3.54E+06 |
| SHP_gp_sali_4_ | **EIIIIIAL** | 28.6 |  |  | 28.6 | 6.72E+04 |
| SHP_gp_sali_3_ | **CIYTIVGGV** | 24.4 |  |  |  |  |
| SHP_Sthermo_12_ | **DIIIIVGG** | 26.4 |  |  |  |  |

^1^Retention time (min)

^2^N.D, not detected

^3^Gray boxes, the SHP encoding gene is not present in the genome

^4^Extracted Ion Chromatogram, area under the curve of the ion current signal

**TABLE S15 :** Detection of natural SHPs by LC-HRMS/MS in the raw supernatant of *S. thermophilus* strain CIRM30

| **Peptide** | **Mature sequence** | **RT^1^ of synthetic standard** | **CIRM30^2^** | |
| --- | --- | --- | --- | --- |
|  |  |  | RT^1^ | EIC^3^ |
| SHP_gp_sali_5_ | **ESIIVIAVG** | 25.3 | 25.3 | 2.48E+06 |
| SHP_Sthermo_6_ | **DIIIFPPFG** | 29.8 |  |  |
| SHP_gp_sali_6_ | **EGIIVIVVG** | 26.2 |  |  |
| SHP_gp_sali_7_ | **EGIIVILVG** | 26.0 | N.D | N.D |
| SHP_Sthermo_13_ | **EGIIVIGVG** | 25.2 | 25.1 | 2.17E+06 |
| SHP_gp_sali_4_ | **EIIIIIAL** | 28.6 |  |  |
| SHP_gp_sali_3_ | **CIYIIVGGV** | NA | 24.32 | 1.03E+05 |
| SHP_Sthermo_12_ | **DIIIIVGG** | 26.4 |  |  |

^1^Retention time (min) ; NA, not available

^2^Gray boxes, the SHP encoding gene is not present in the genome

^3^Extracted Ion Chromatogram, area under the curve of the ion current signal
