# Supplementary material for: RRNPP quorum-sensing repertoires in the salivarius group genomes: overrepresentation and synchronous activation of SHP/Rgg systems in Streptococcus thermophilus
Source: J Bacteriol. 2025 Aug 26;207(9):e00231-25. doi: 10.1128/jb.00231-25 (PMC12445099; doi:10.1128/jb.00231-25)
Supplement: Supplemental figures — Figures S1 to S18. [file jb.00231-25-s0002.ppt]

## Slide 1
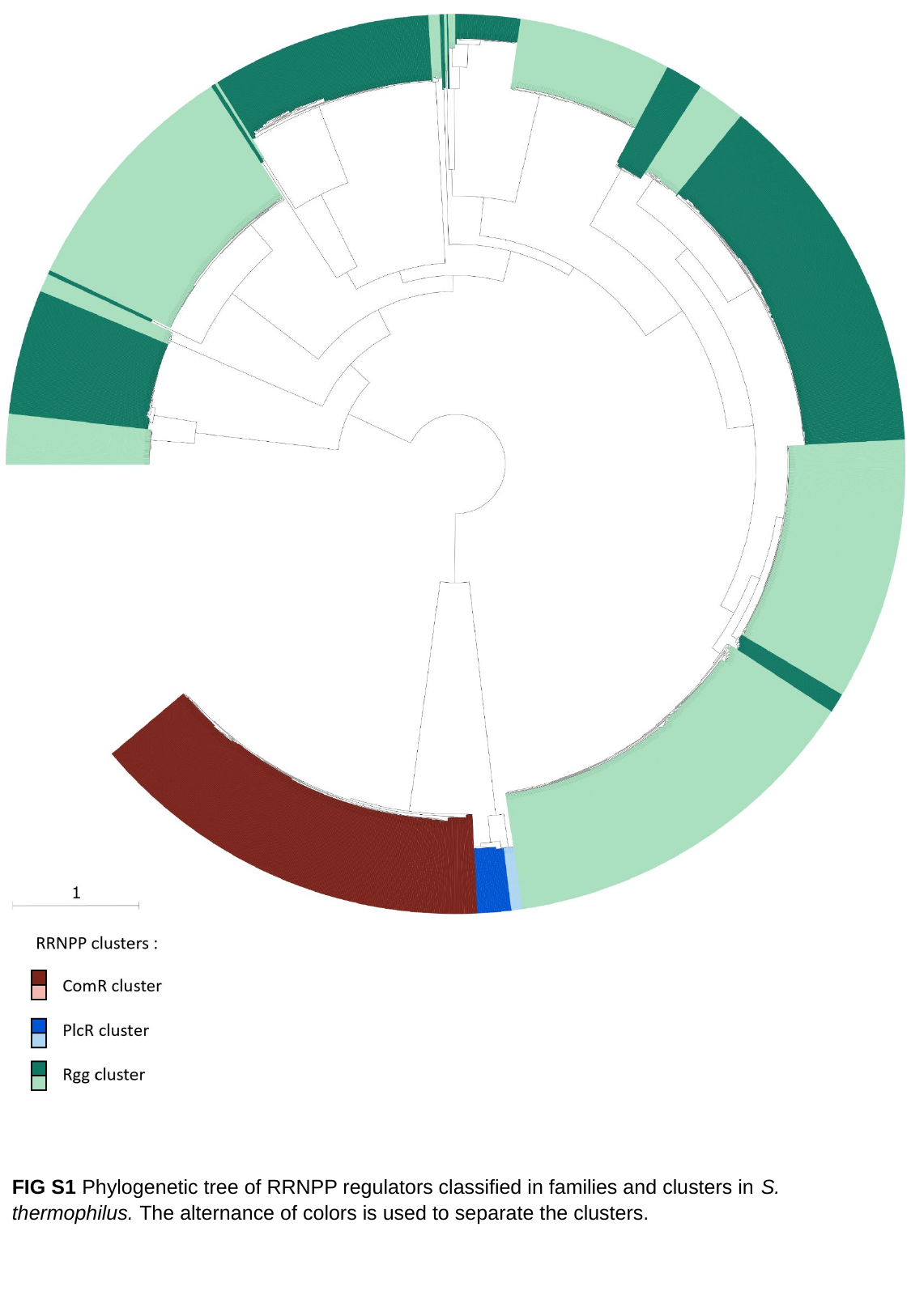

FIG S1 Phylogenetic tree of RRNPP regulators classified in families and clusters in S. thermophilus. The alternance of colors is used to separate the clusters.

## Slide 2
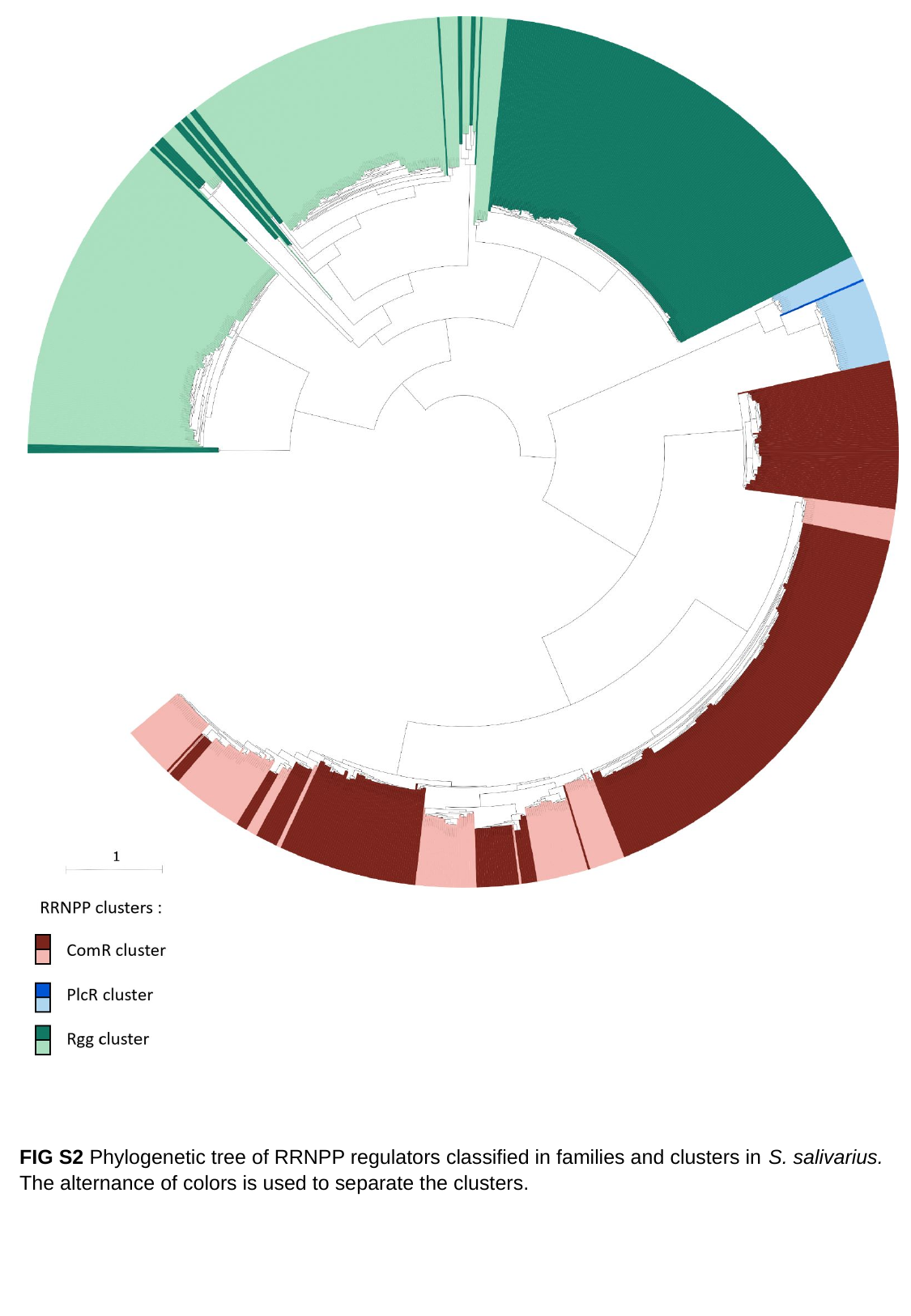

#
FIG S2 Phylogenetic tree of RRNPP regulators classified in families and clusters in S. salivarius. The alternance of colors is used to separate the clusters.

## Slide 3
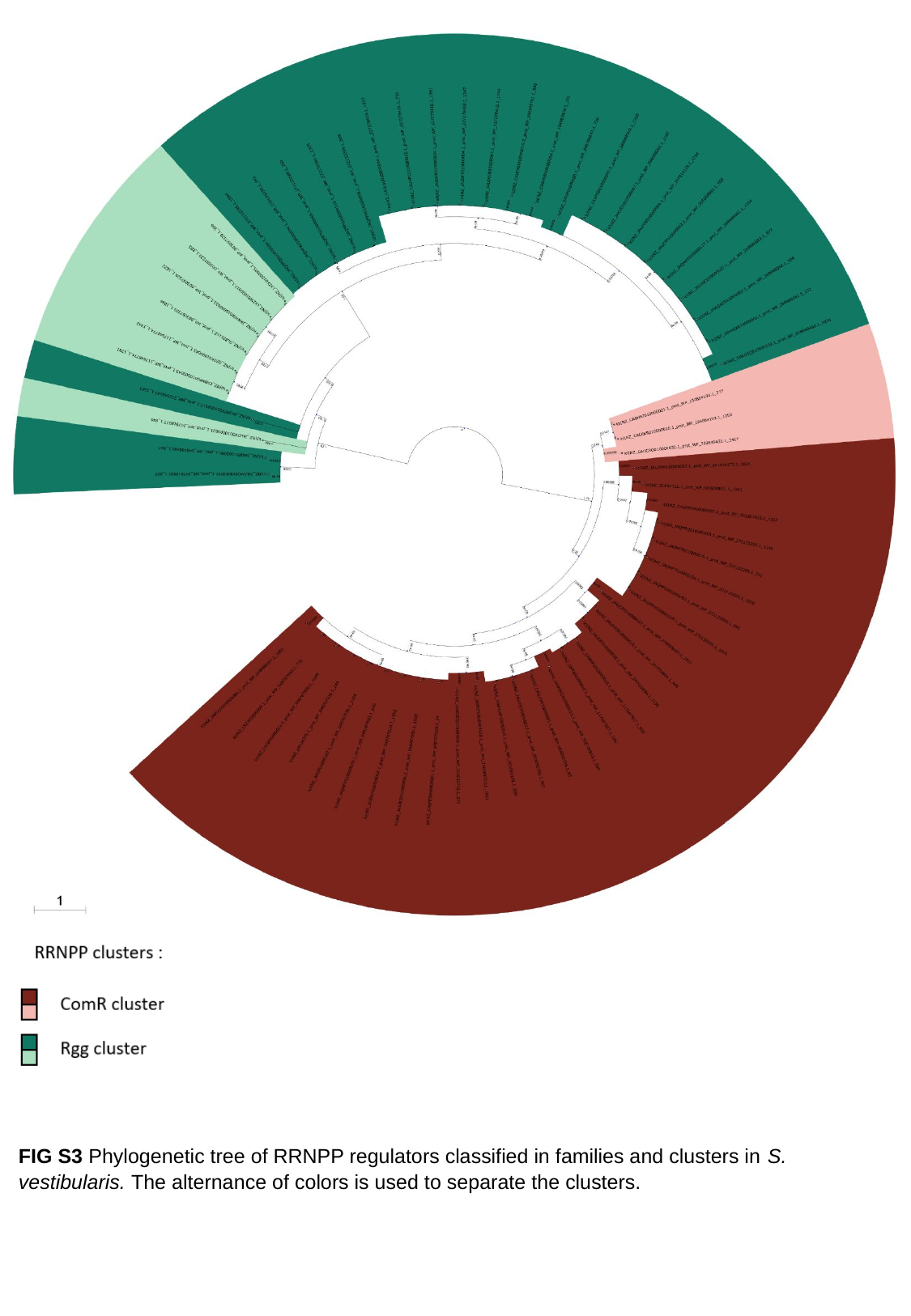

#
FIG S3 Phylogenetic tree of RRNPP regulators classified in families and clusters in S. vestibularis. The alternance of colors is used to separate the clusters.

## Slide 4
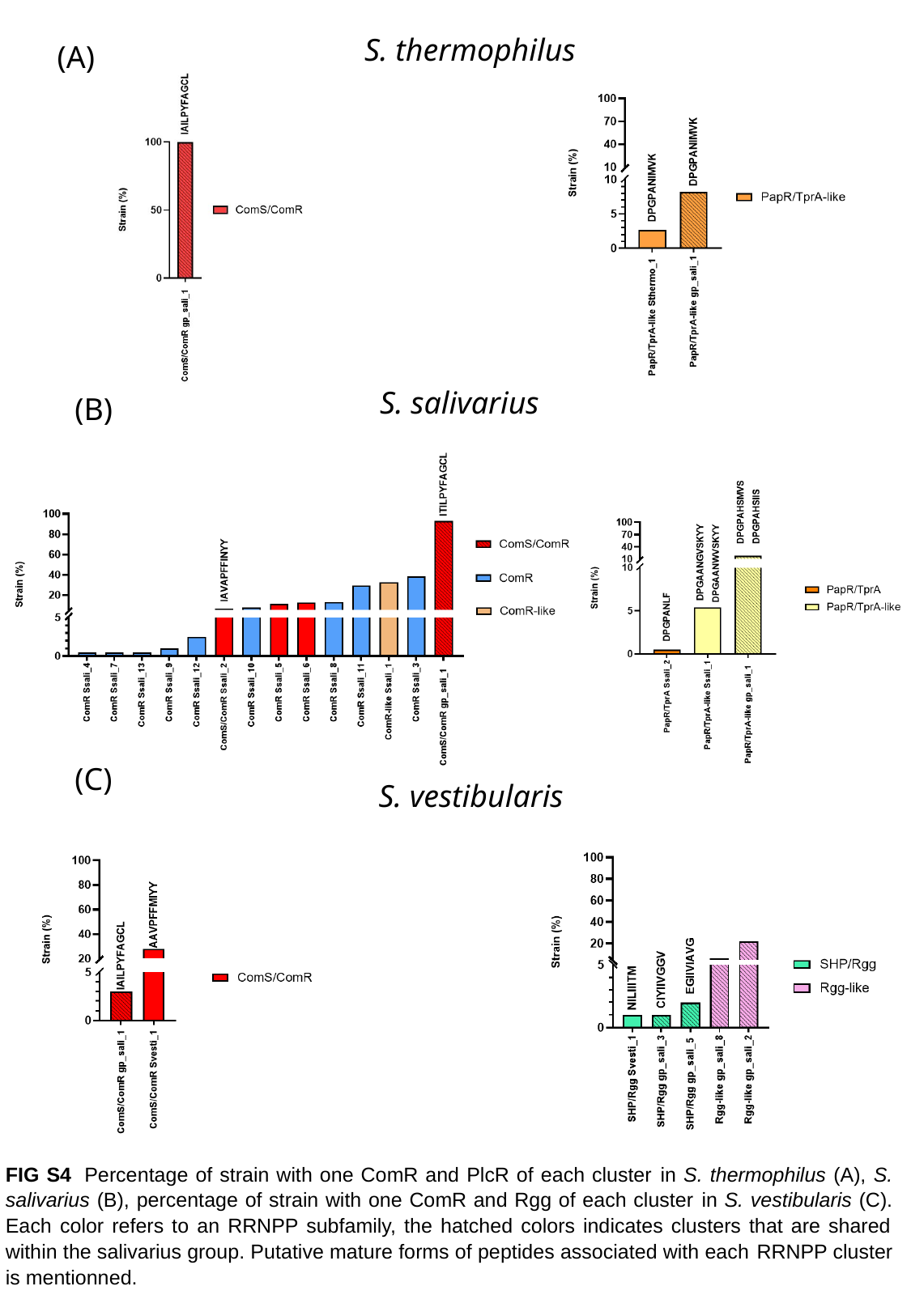

S. thermophilus
(A)
S. salivarius
(B)
(C)
S. vestibularis
FIG S4  Percentage of strain with one ComR and PlcR of each cluster in S. thermophilus (A), S. salivarius (B), percentage of strain with one ComR and Rgg of each cluster in S. vestibularis (C). Each color refers to an RRNPP subfamily, the hatched colors indicates clusters that are shared within the salivarius group. Putative mature forms of peptides associated with each RRNPP cluster is mentionned.

## Slide 5
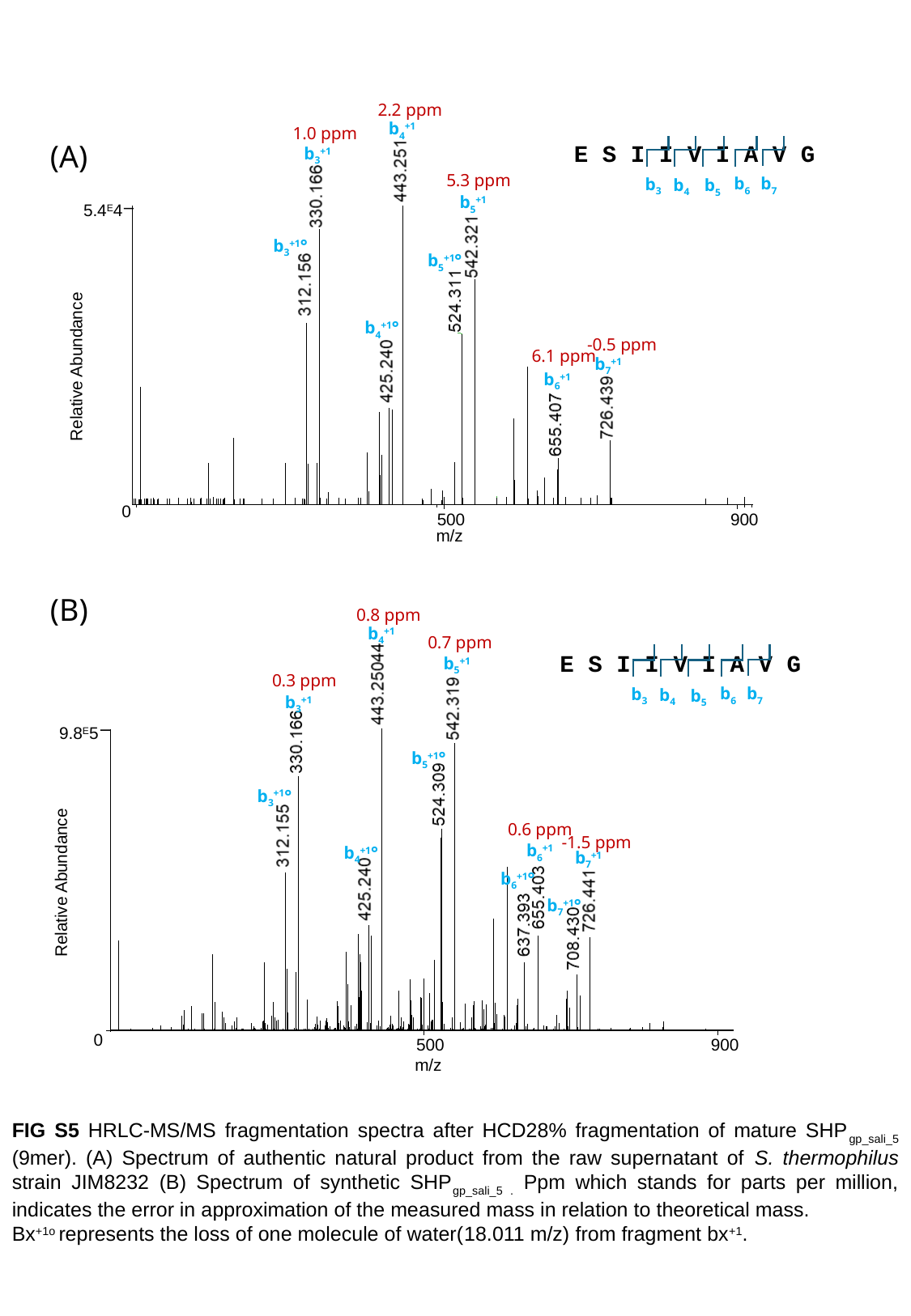

2.2 ppm
b4+1
b3+1
b5+1
5.4E4
b3+1°
b5+1°
b4+1°
b7+1
b6+1
0
500
900
m/z
1.0 ppm
E S I I V I A V G
b7
b6
b3
b4
b5
5.3 ppm
-0.5 ppm
6.1 ppm
(A)
Relative Abundance
(B)
0.8 ppm
b4+1
b5+1
b3+1
9.8E5
b5+1°
b3+1°
b6+1
b4+1°
b7+1
b6+1°
b7+1°
0
500
900
m/z
0.7 ppm
E S I I V I A V G
b7
b6
b3
b4
b5
0.3 ppm
0.6 ppm
-1.5 ppm
Relative Abundance
FIG S5 HRLC-MS/MS fragmentation spectra after HCD28% fragmentation of mature SHPgp_sali_5 (9mer). (A) Spectrum of authentic natural product from the raw supernatant of S. thermophilus strain JIM8232 (B) Spectrum of synthetic SHPgp_sali_5 . Ppm which stands for parts per million, indicates the error in approximation of the measured mass in relation to theoretical mass.
Bx+1o represents the loss of one molecule of water(18.011 m/z) from fragment bx+1.

## Slide 6
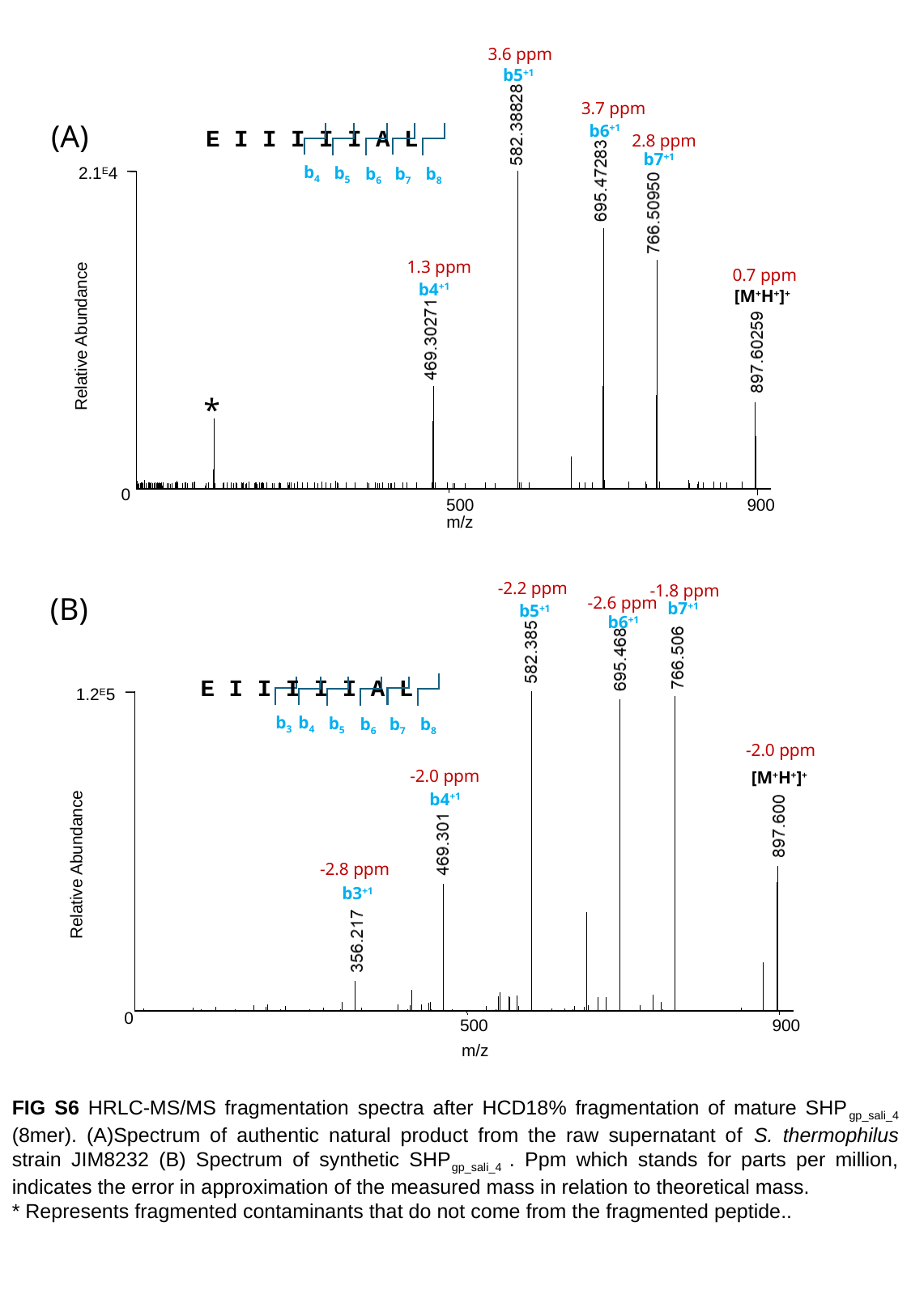

3.6 ppm
b5+1
3.7 ppm
E I I I I I A L
b4
b5
b8
b7
b6
b6+1
2.8 ppm
b7+1
2.1E4
1.3 ppm
0.7 ppm
b4+1
[M+H+]+
0
500
900
m/z
(A)
Relative Abundance
*
-2.2 ppm
-1.8 ppm
-2.6 ppm
b7+1
b5+1
b6+1
E I I I I I A L
b4
b5
b7
b8
b6
b3
1.2E5
-2.0 ppm
-2.0 ppm
[M+H+]+
b4+1
-2.8 ppm
b3+1
0
500
900
m/z
(B)
Relative Abundance
FIG S6 HRLC-MS/MS fragmentation spectra after HCD18% fragmentation of mature SHPgp_sali_4 (8mer). (A)Spectrum of authentic natural product from the raw supernatant of S. thermophilus strain JIM8232 (B) Spectrum of synthetic SHPgp_sali_4 . Ppm which stands for parts per million, indicates the error in approximation of the measured mass in relation to theoretical mass.
* Represents fragmented contaminants that do not come from the fragmented peptide..

## Slide 7
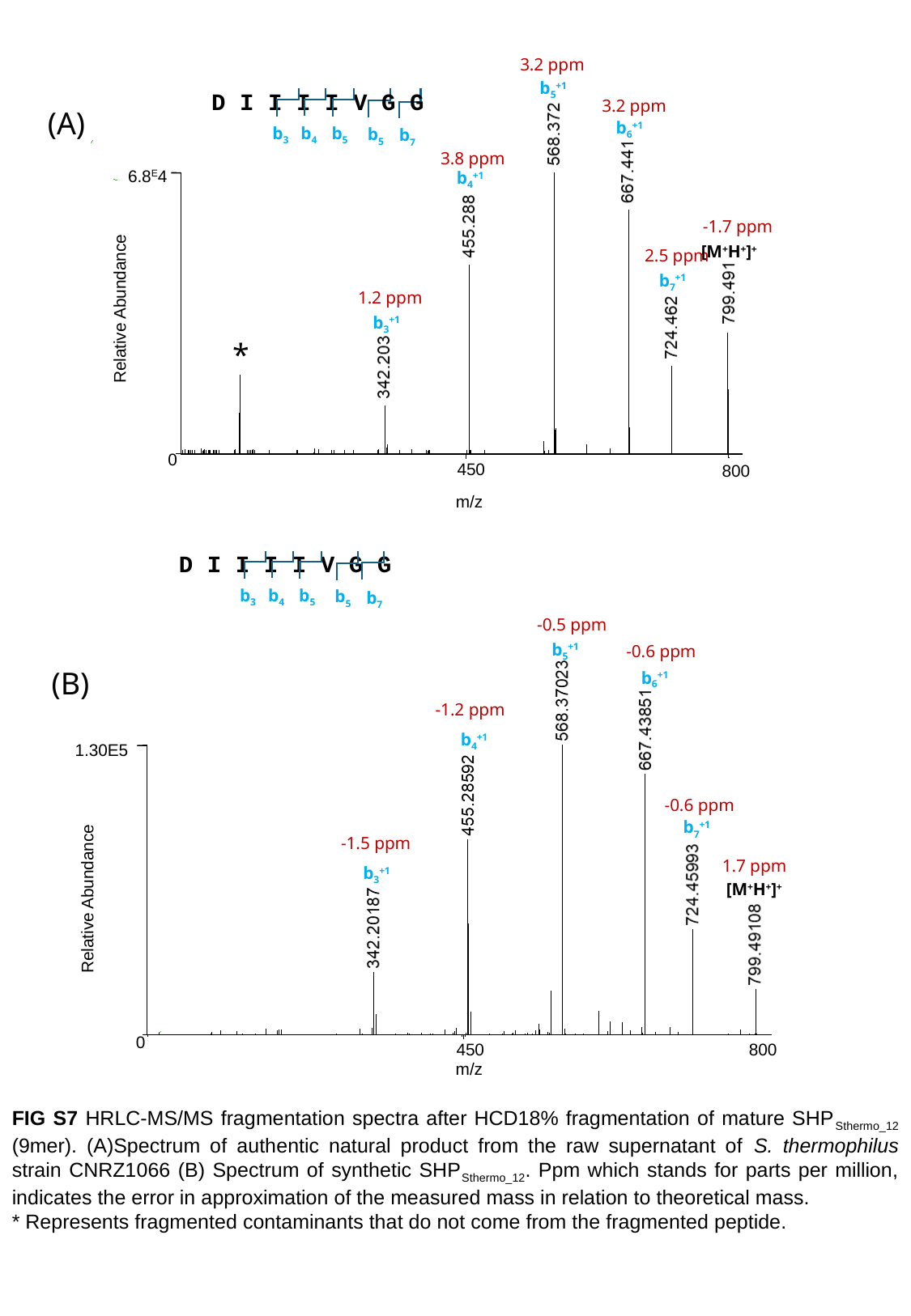

3.2 ppm
b5+1
D I I I I V G G
b4
b3
b5
b5
b7
3.2 ppm
b6+1
3.8 ppm
b4+1
6.8E4
-1.7 ppm
[M+H+]+
2.5 ppm
b7+1
1.2 ppm
b3+1
0
450
800
m/z
(A)
Relative Abundance
*
D I I I I V G G
b4
b3
b5
b5
b7
-0.5 ppm
b5+1
-0.6 ppm
(B)
b6+1
-1.2 ppm
b4+1
1.30E5
-0.6 ppm
b7+1
-1.5 ppm
1.7 ppm
b3+1
[M+H+]+
Relative Abundance
0
450
800
m/z
FIG S7 HRLC-MS/MS fragmentation spectra after HCD18% fragmentation of mature SHPSthermo_12 (9mer). (A)Spectrum of authentic natural product from the raw supernatant of S. thermophilus strain CNRZ1066 (B) Spectrum of synthetic SHPSthermo_12. Ppm which stands for parts per million, indicates the error in approximation of the measured mass in relation to theoretical mass.
* Represents fragmented contaminants that do not come from the fragmented peptide.

## Slide 8
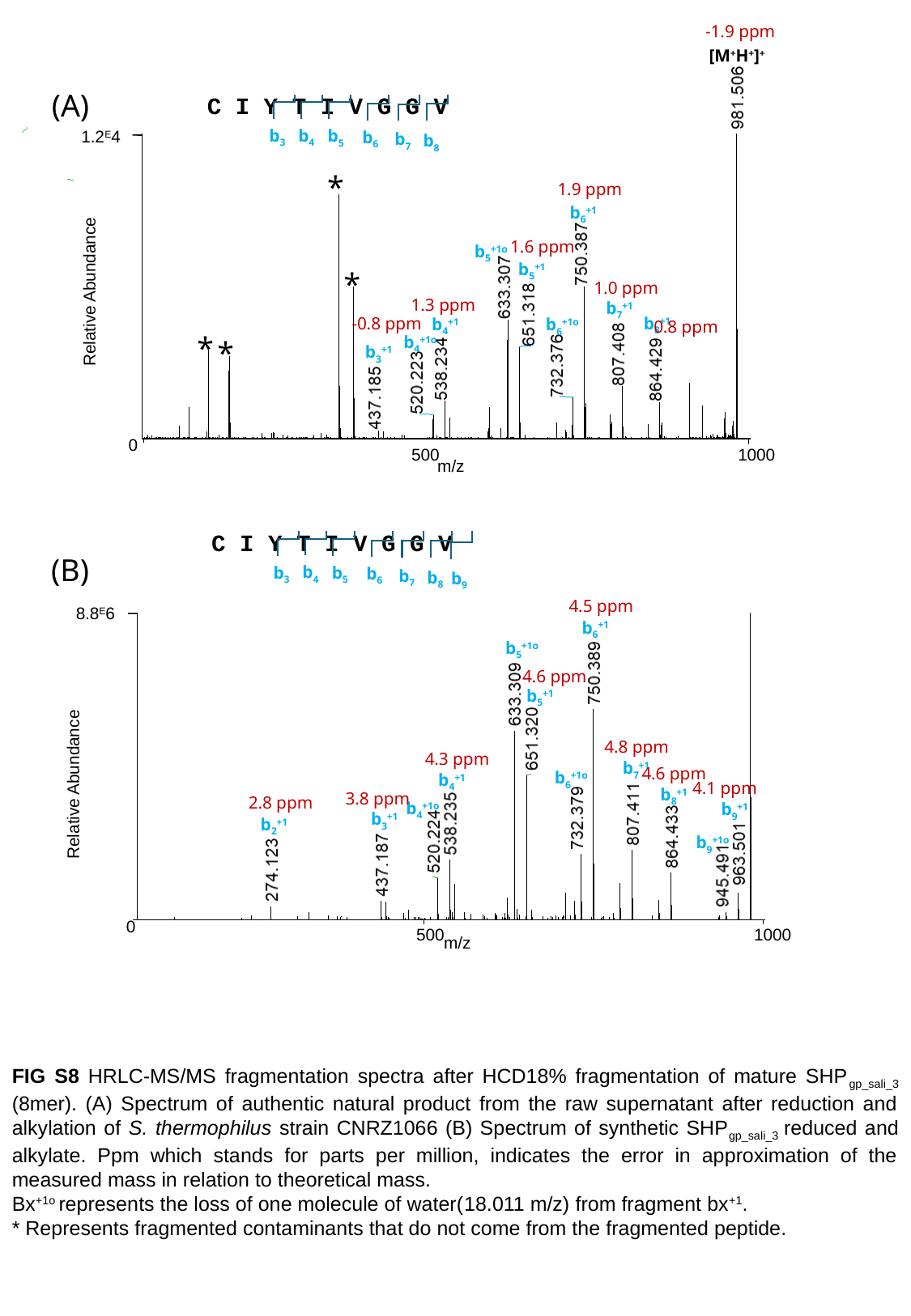

-1.9 ppm
[M+H+]+
C I Y T I V G G V
b4
b3
b5
b6
b7
b8
1.2E4
1.9 ppm
b6+1
1.6 ppm
b5+1o
b5+1
1.0 ppm
1.3 ppm
b7+1
b8+1
-0.8 ppm
b4+1
b6+1o
0.8 ppm
b4+1o
b3+1
0
500
1000
m/z
(A)
*
*
Relative Abundance
*
*
C I Y T I V G G V
b4
b3
b5
b6
b7
b8
b9
4.5 ppm
8.8E6
b6+1
b5+1o
4.6 ppm
b5+1
4.8 ppm
4.3 ppm
b7+1
4.6 ppm
b6+1o
b4+1
4.1 ppm
b8+1
3.8 ppm
2.8 ppm
b4+1o
b9+1
b3+1
b2+1
b9+1o
0
500
1000
m/z
(B)
Relative Abundance
FIG S8 HRLC-MS/MS fragmentation spectra after HCD18% fragmentation of mature SHPgp_sali_3 (8mer). (A) Spectrum of authentic natural product from the raw supernatant after reduction and alkylation of S. thermophilus strain CNRZ1066 (B) Spectrum of synthetic SHPgp_sali_3 reduced and alkylate. Ppm which stands for parts per million, indicates the error in approximation of the measured mass in relation to theoretical mass.
Bx+1o represents the loss of one molecule of water(18.011 m/z) from fragment bx+1.
* Represents fragmented contaminants that do not come from the fragmented peptide.

## Slide 9
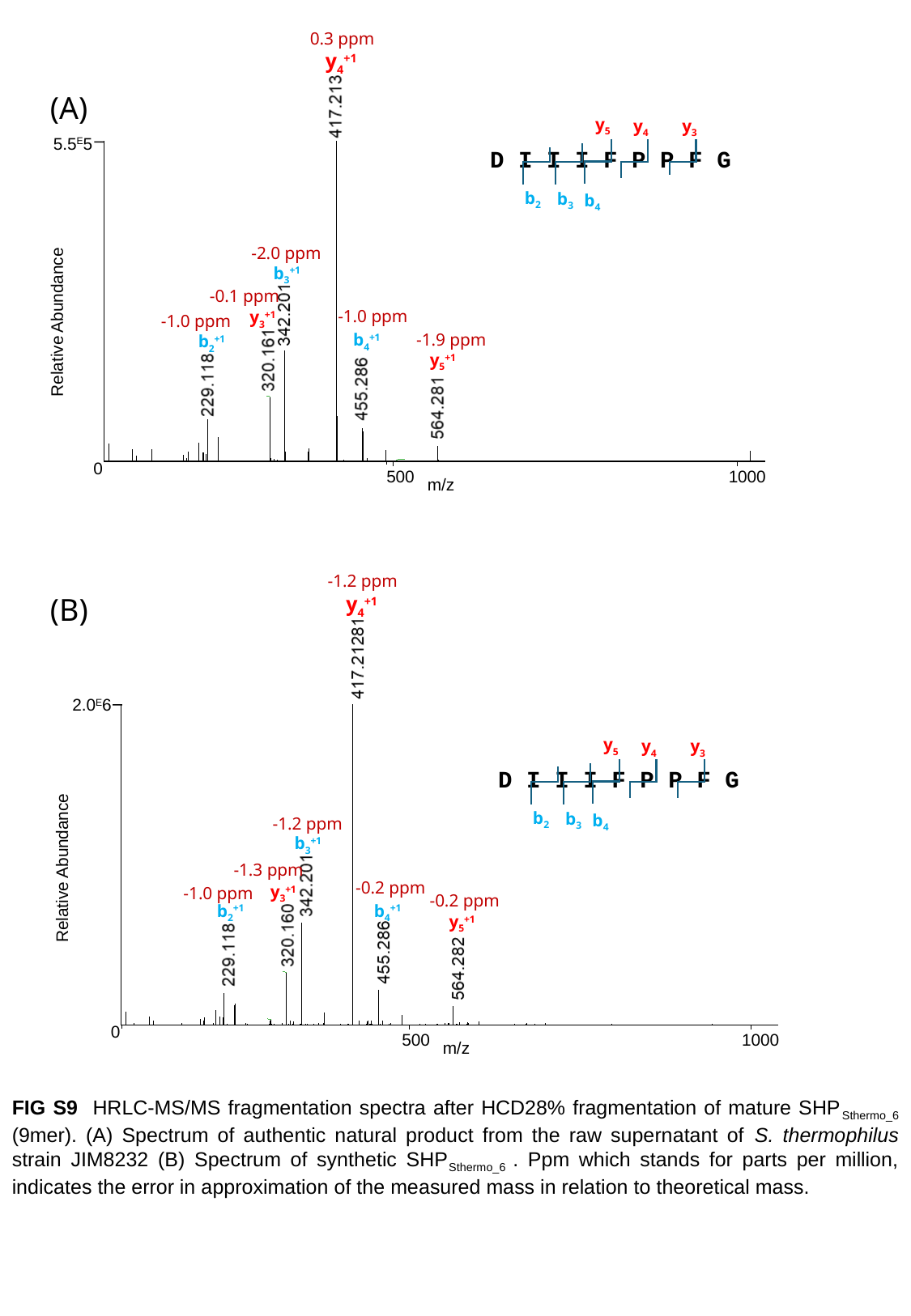

0.3 ppm
y4+1
y5
y3
y4
D I I I F P P F G
b2
b3
b4
5.5E5
-2.0 ppm
b3+1
-0.1 ppm
-1.0 ppm
y3+1
-1.0 ppm
-1.9 ppm
b4+1
b2+1
y5+1
0
500
1000
m/z
(A)
Relative Abundance
-1.2 ppm
y4+1
2.0E6
-1.2 ppm
b3+1
-1.3 ppm
-0.2 ppm
y3+1
-1.0 ppm
-0.2 ppm
b2+1
b4+1
y5+1
0
500
1000
m/z
y5
y3
y4
D I I I F P P F G
b2
b3
b4
(B)
Relative Abundance
FIG S9 HRLC-MS/MS fragmentation spectra after HCD28% fragmentation of mature SHPSthermo_6 (9mer). (A) Spectrum of authentic natural product from the raw supernatant of S. thermophilus strain JIM8232 (B) Spectrum of synthetic SHPSthermo_6 . Ppm which stands for parts per million, indicates the error in approximation of the measured mass in relation to theoretical mass.

## Slide 10
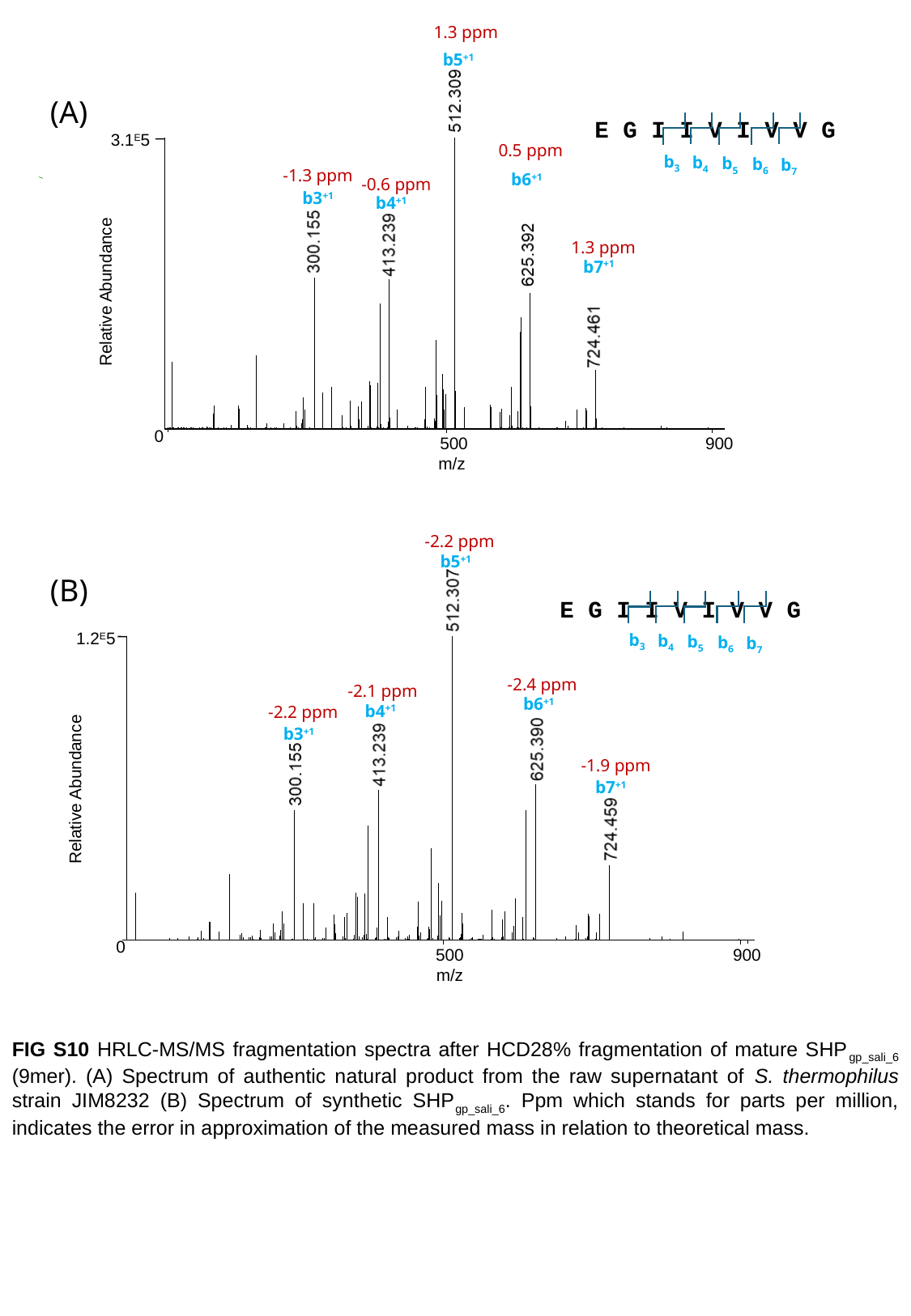

1.3 ppm
b5+1
E G I I V I V V G
b3
b4
b5
b6
b7
3.1E5
0.5 ppm
-1.3 ppm
b6+1
-0.6 ppm
b3+1
b4+1
1.3 ppm
b7+1
0
500
900
m/z
(A)
Relative Abundance
-2.2 ppm
b5+1
E G I I V I V V G
b3
b4
b5
b6
b7
1.2E5
-2.4 ppm
-2.1 ppm
b6+1
b4+1
-2.2 ppm
b3+1
-1.9 ppm
b7+1
0
500
900
m/z
(B)
Relative Abundance
FIG S10 HRLC-MS/MS fragmentation spectra after HCD28% fragmentation of mature SHPgp_sali_6 (9mer). (A) Spectrum of authentic natural product from the raw supernatant of S. thermophilus strain JIM8232 (B) Spectrum of synthetic SHPgp_sali_6. Ppm which stands for parts per million, indicates the error in approximation of the measured mass in relation to theoretical mass.

## Slide 11
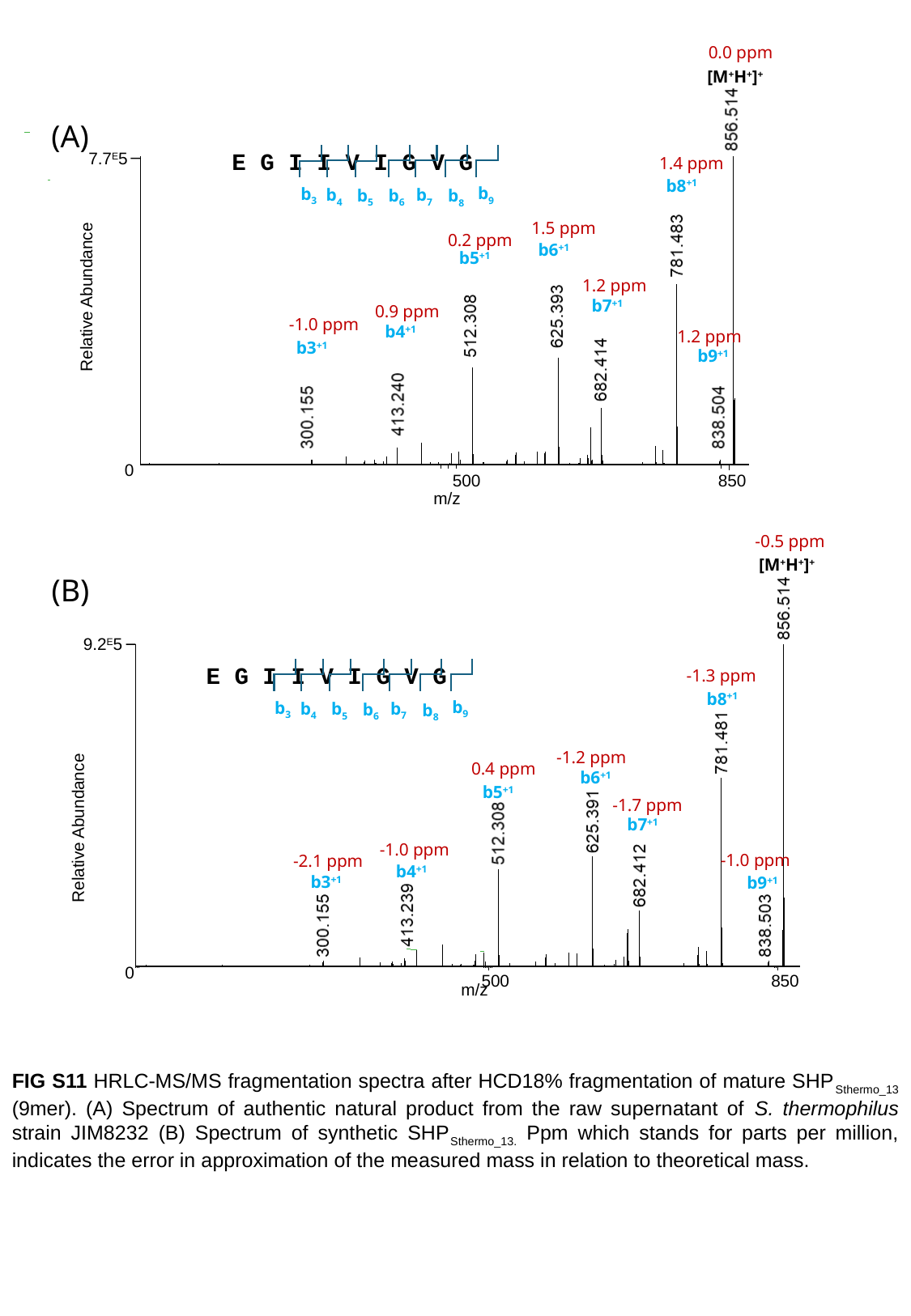

0.0 ppm
[M+H+]+
E G I I V I G V G
b9
b3
b7
b4
b5
b6
b8
1.4 ppm
7.7E5
b8+1
1.5 ppm
0.2 ppm
b6+1
b5+1
1.2 ppm
b7+1
0.9 ppm
-1.0 ppm
b4+1
1.2 ppm
b3+1
b9+1
0
500
850
m/z
(A)
Relative Abundance
-0.5 ppm
[M+H+]+
9.2E5
E G I I V I G V G
b9
b3
b4
b7
b5
b6
b8
-1.3 ppm
b8+1
-1.2 ppm
0.4 ppm
b6+1
b5+1
-1.7 ppm
b7+1
-1.0 ppm
-1.0 ppm
-2.1 ppm
b4+1
b3+1
b9+1
0
500
850
m/z
(B)
Relative Abundance
FIG S11 HRLC-MS/MS fragmentation spectra after HCD18% fragmentation of mature SHPSthermo_13 (9mer). (A) Spectrum of authentic natural product from the raw supernatant of S. thermophilus strain JIM8232 (B) Spectrum of synthetic SHPSthermo_13. Ppm which stands for parts per million, indicates the error in approximation of the measured mass in relation to theoretical mass.

## Slide 12
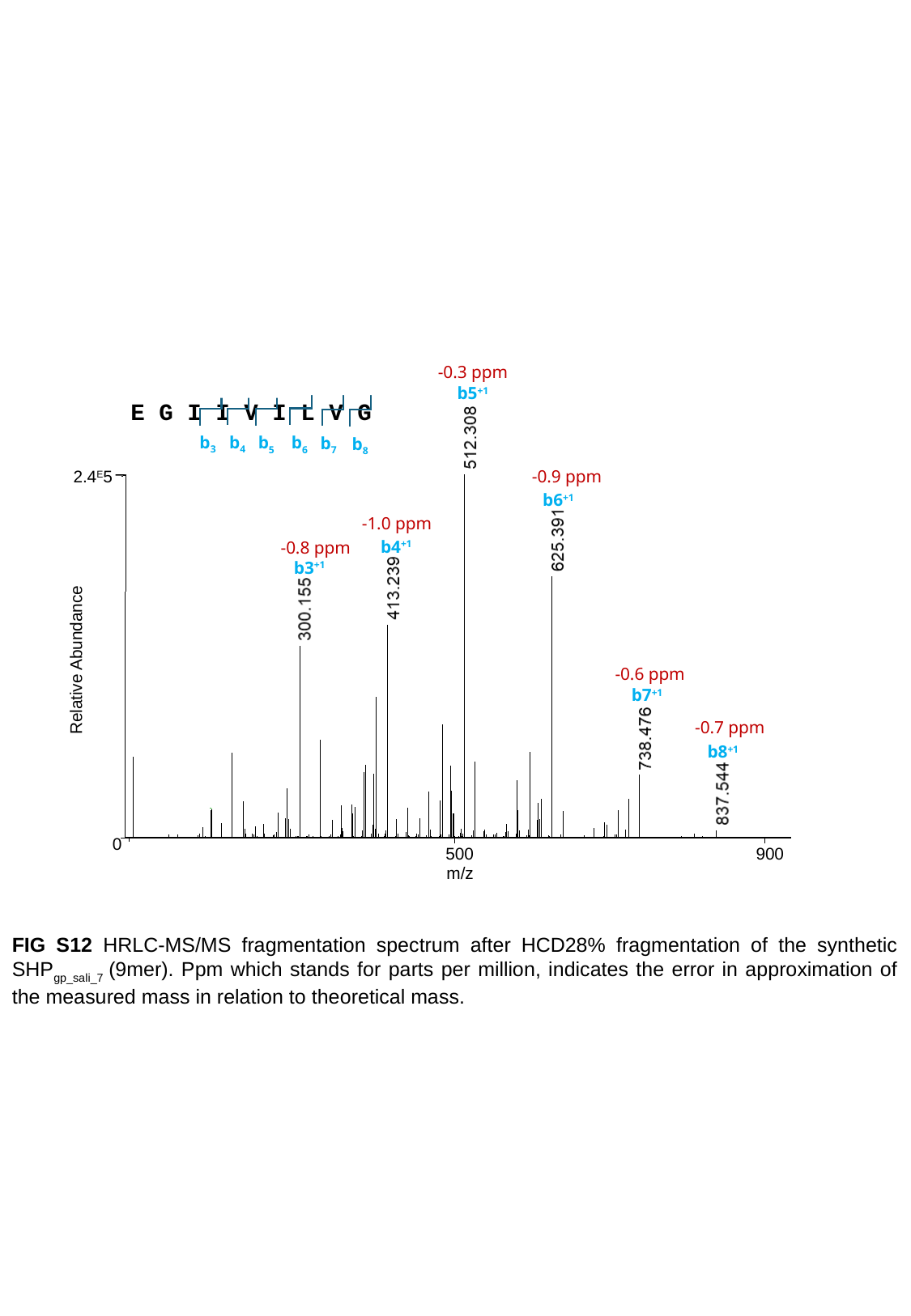

-0.3 ppm
E G I I V I L V G
b4
b3
b5
b6
b7
b8
b5+1
-0.9 ppm
2.4E5
b6+1
-1.0 ppm
b4+1
-0.8 ppm
b3+1
-0.6 ppm
b7+1
-0.7 ppm
b8+1
0
500
900
m/z
Relative Abundance
FIG S12 HRLC-MS/MS fragmentation spectrum after HCD28% fragmentation of the synthetic SHPgp_sali_7 (9mer). Ppm which stands for parts per million, indicates the error in approximation of the measured mass in relation to theoretical mass.

## Slide 13
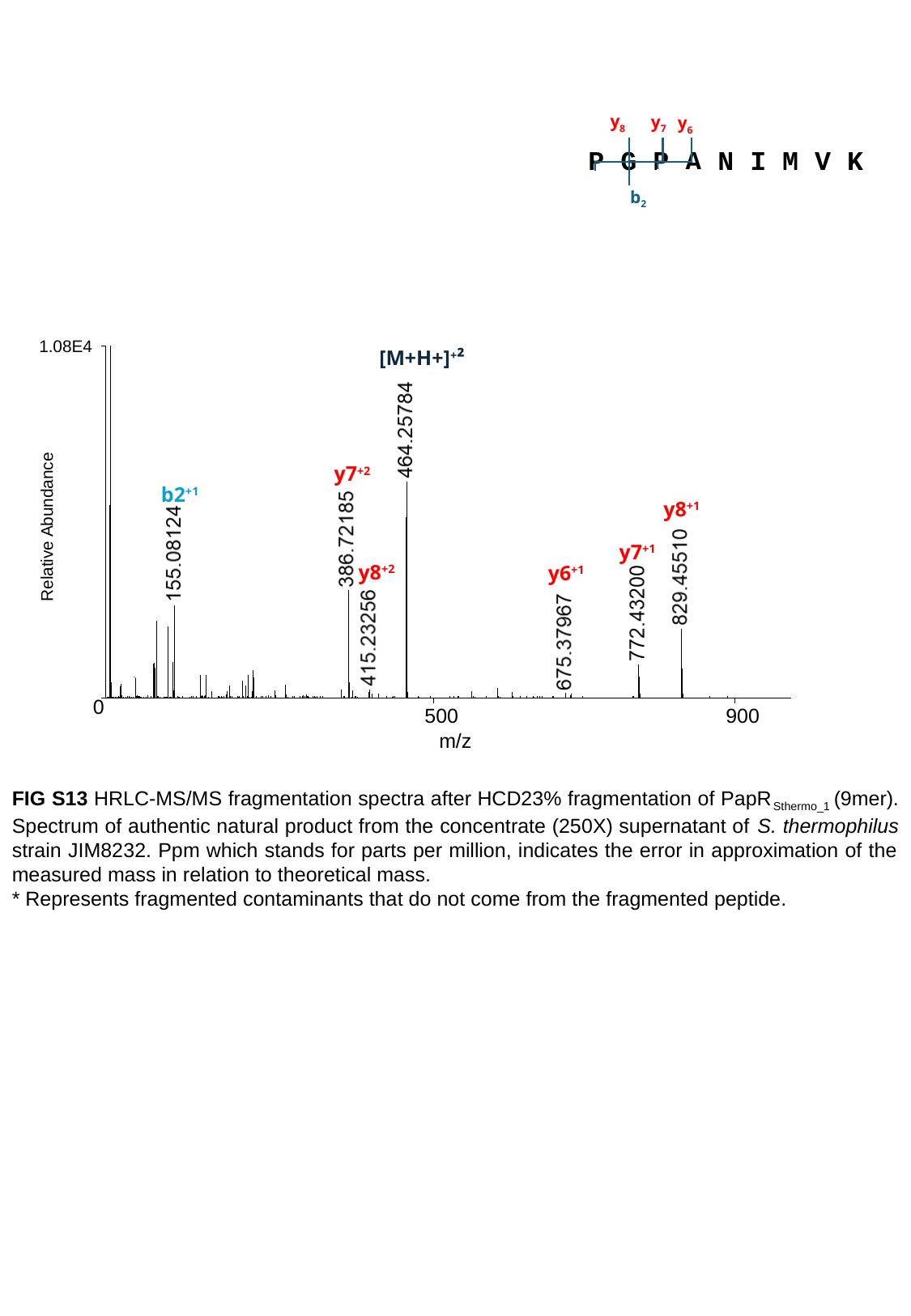

y8
y7
y6
P G P A N I M V K
b2
1.08E4
[M+H+]+²
y7+2
b2+1
y8+1
Relative Abundance
y7+1
y8+2
y6+1
0
500
900
m/z
FIG S13 HRLC-MS/MS fragmentation spectra after HCD23% fragmentation of PapRSthermo_1 (9mer). Spectrum of authentic natural product from the concentrate (250X) supernatant of S. thermophilus strain JIM8232. Ppm which stands for parts per million, indicates the error in approximation of the measured mass in relation to theoretical mass.
* Represents fragmented contaminants that do not come from the fragmented peptide.

## Slide 14
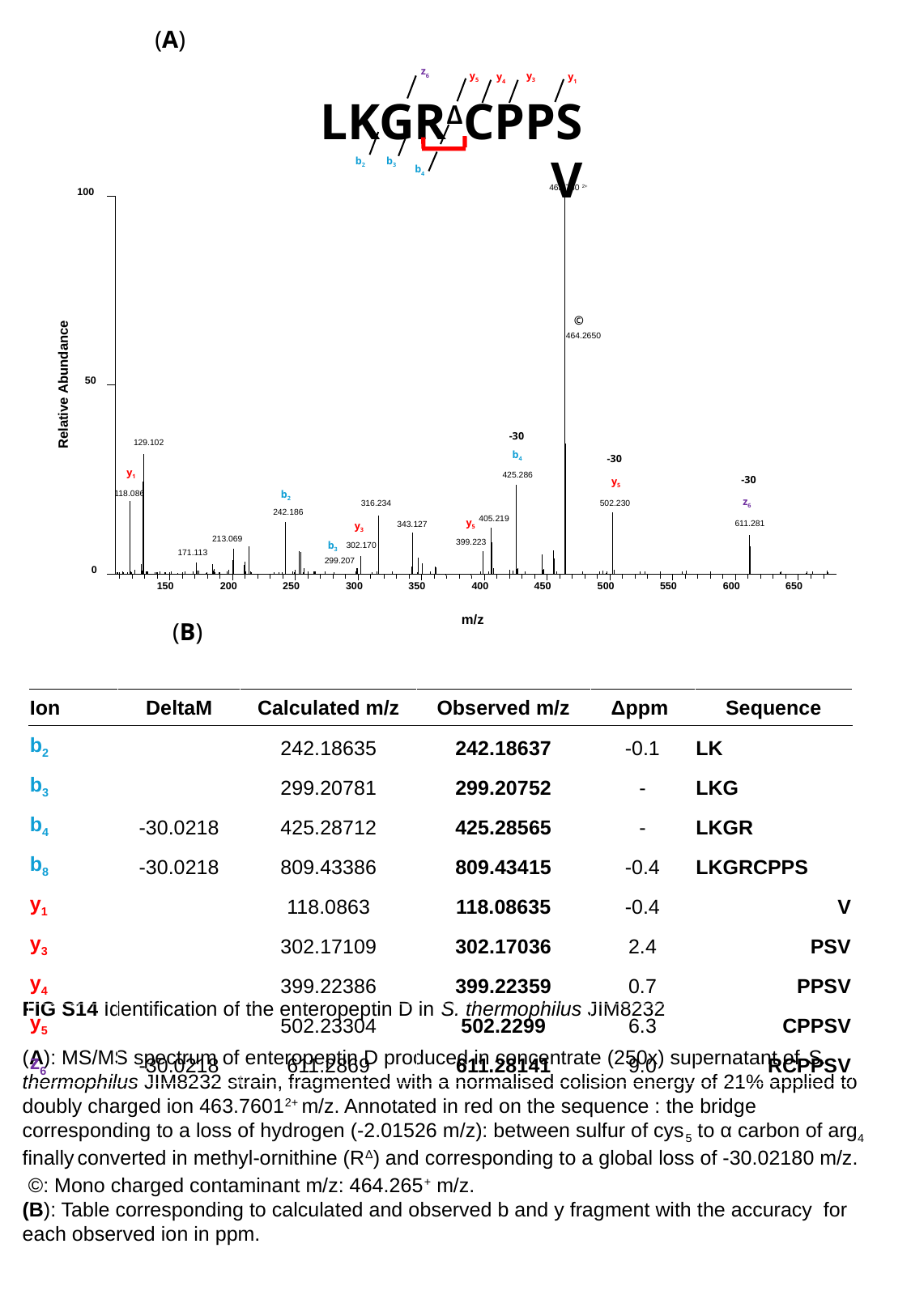

(A)
z6
y5
y3
y4
y1
LKGRΔCPPSV
b2
b3
b4
463.760 2+
100
©
464.2650
50
Relative Abundance
-30
129.102
b4
-30
y1
-30
y5
425.286
b2
118.086
z6
316.234
502.230
242.186
y5
405.219
y3
611.281
343.127
b3
213.069
399.223
302.170
171.113
299.207
0
150
200
250
300
350
400
450
500
550
600
650
m/z
(B)
| Ion | DeltaM | Calculated m/z | Observed m/z | Δppm | Sequence |
| --- | --- | --- | --- | --- | --- |
| b2 | | 242.18635 | 242.18637 | -0.1 | LK |
| b3 | | 299.20781 | 299.20752 | - | LKG |
| b4 | -30.0218 | 425.28712 | 425.28565 | - | LKGR |
| b8 | -30.0218 | 809.43386 | 809.43415 | -0.4 | LKGRCPPS |
| y1 | | 118.0863 | 118.08635 | -0.4 | V |
| y3 | | 302.17109 | 302.17036 | 2.4 | PSV |
| y4 | | 399.22386 | 399.22359 | 0.7 | PPSV |
| y5 | | 502.23304 | 502.2299 | 6.3 | CPPSV |
| z6 | -30.0218 | 611.2869 | 611.28141 | 9.0 | RCPPSV |
FIG S14 Identification of the enteropeptin D in S. thermophilus JIM8232
(A): MS/MS spectrum of enteropeptin D produced in concentrate (250x) supernatant of S. thermophilus JIM8232 strain, fragmented with a normalised colision energy of 21% applied to doubly charged ion 463.76012+ m/z. Annotated in red on the sequence : the bridge corresponding to a loss of hydrogen (-2.01526 m/z): between sulfur of cys5 to α carbon of arg4 finally converted in methyl-ornithine (RΔ) and corresponding to a global loss of -30.02180 m/z.
 ©: Mono charged contaminant m/z: 464.265+ m/z.
(B): Table corresponding to calculated and observed b and y fragment with the accuracy for each observed ion in ppm.

## Slide 15
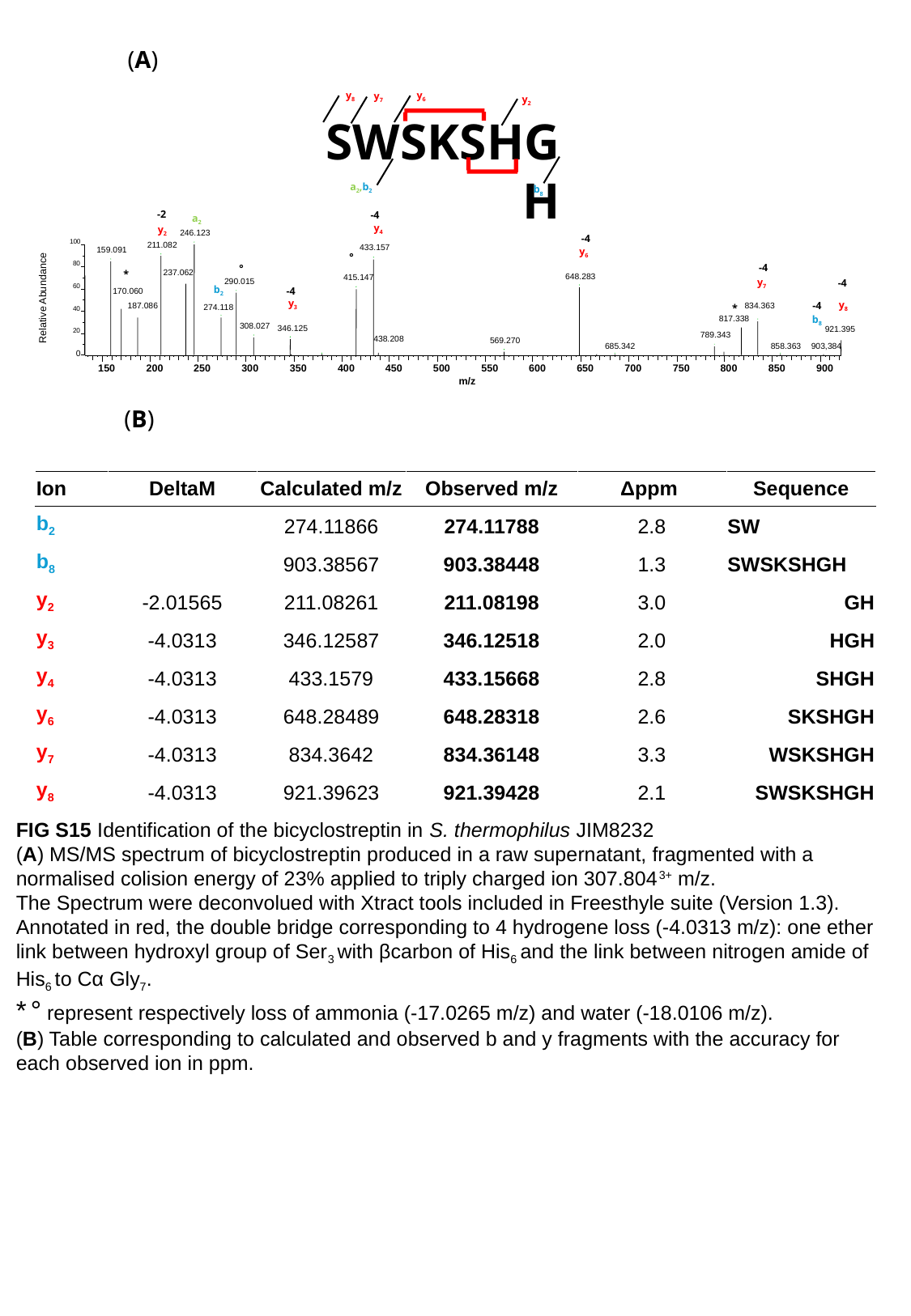

(A)
y8
y6
y7
y2
SWSKSHGH
a2,b2
b8
-2
-4
a2
y4
y2
-4
246.123
100
y6
211.082
433.157
159.091
°
-4
°
80
*
237.062
y7
648.283
-4
415.147
290.015
b2
-4
60
170.060
y3
y8
-4
*
834.363
187.086
274.118
40
b8
817.338
308.027
346.125
921.395
20
789.343
438.208
569.270
685.342
858.363
903,384
0
150
200
250
300
350
400
450
500
550
600
650
700
750
800
850
900
m/z
Relative Abundance
(B)
| Ion | DeltaM | Calculated m/z | Observed m/z | Δppm | Sequence |
| --- | --- | --- | --- | --- | --- |
| b2 | | 274.11866 | 274.11788 | 2.8 | SW |
| b8 | | 903.38567 | 903.38448 | 1.3 | SWSKSHGH |
| y2 | -2.01565 | 211.08261 | 211.08198 | 3.0 | GH |
| y3 | -4.0313 | 346.12587 | 346.12518 | 2.0 | HGH |
| y4 | -4.0313 | 433.1579 | 433.15668 | 2.8 | SHGH |
| y6 | -4.0313 | 648.28489 | 648.28318 | 2.6 | SKSHGH |
| y7 | -4.0313 | 834.3642 | 834.36148 | 3.3 | WSKSHGH |
| y8 | -4.0313 | 921.39623 | 921.39428 | 2.1 | SWSKSHGH |
FIG S15 Identification of the bicyclostreptin in S. thermophilus JIM8232
(A) MS/MS spectrum of bicyclostreptin produced in a raw supernatant, fragmented with a normalised colision energy of 23% applied to triply charged ion 307.8043+ m/z.
The Spectrum were deconvolued with Xtract tools included in Freesthyle suite (Version 1.3).
Annotated in red, the double bridge corresponding to 4 hydrogene loss (-4.0313 m/z): one ether link between hydroxyl group of Ser3 with βcarbon of His6 and the link between nitrogen amide of His6 to Cα Gly7.
* ° represent respectively loss of ammonia (-17.0265 m/z) and water (-18.0106 m/z).
(B) Table corresponding to calculated and observed b and y fragments with the accuracy for each observed ion in ppm.

## Slide 16
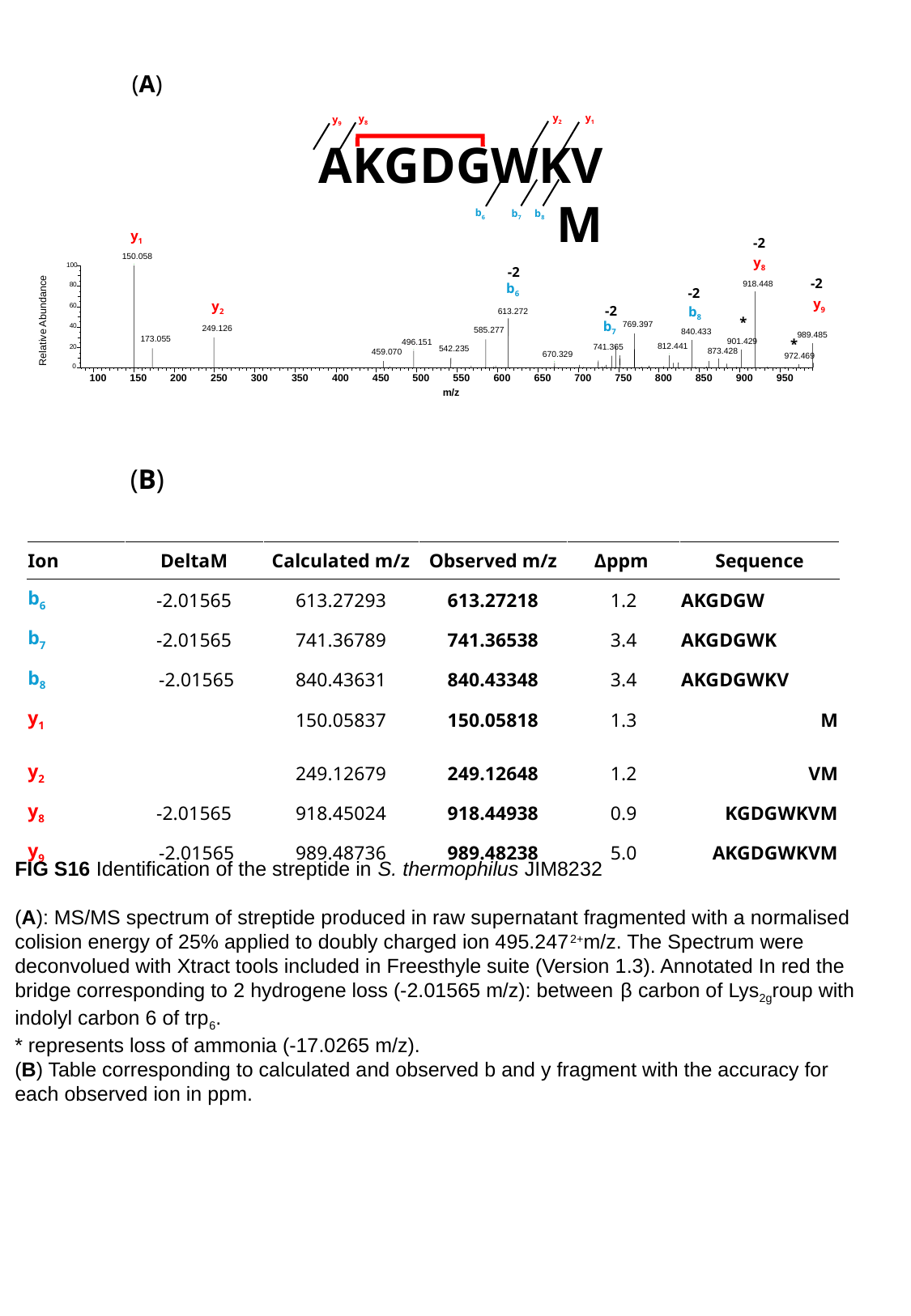

(A)
y2
y1
y8
y9
AKGDGWKVM
b6
b7
b8
y1
-2
y8
150.058
-2
100
-2
b6
-2
918.448
80
y9
y2
-2
b8
60
613.272
*
b7
769.397
40
249.126
585.277
840.433
989.485
*
173.055
901.429
496.151
812.441
741.365
20
542.235
873.428
459.070
670.329
972.469
0
100
150
200
250
300
350
400
450
500
550
600
650
700
750
800
850
900
950
m/z
Relative Abundance
(B)
| Ion | DeltaM | Calculated m/z | Observed m/z | Δppm | Sequence |
| --- | --- | --- | --- | --- | --- |
| b6 | -2.01565 | 613.27293 | 613.27218 | 1.2 | AKGDGW |
| b7 | -2.01565 | 741.36789 | 741.36538 | 3.4 | AKGDGWK |
| b8 | -2.01565 | 840.43631 | 840.43348 | 3.4 | AKGDGWKV |
| y1 | | 150.05837 | 150.05818 | 1.3 | M |
| y2 | | 249.12679 | 249.12648 | 1.2 | VM |
| y8 | -2.01565 | 918.45024 | 918.44938 | 0.9 | KGDGWKVM |
| y9 | -2.01565 | 989.48736 | 989.48238 | 5.0 | AKGDGWKVM |
FIG S16 Identification of the streptide in S. thermophilus JIM8232
(A): MS/MS spectrum of streptide produced in raw supernatant fragmented with a normalised colision energy of 25% applied to doubly charged ion 495.2472+m/z. The Spectrum were deconvolued with Xtract tools included in Freesthyle suite (Version 1.3). Annotated In red the bridge corresponding to 2 hydrogene loss (-2.01565 m/z): between β carbon of Lys2group with indolyl carbon 6 of trp6.
* represents loss of ammonia (-17.0265 m/z).
(B) Table corresponding to calculated and observed b and y fragment with the accuracy for each observed ion in ppm.

## Slide 17
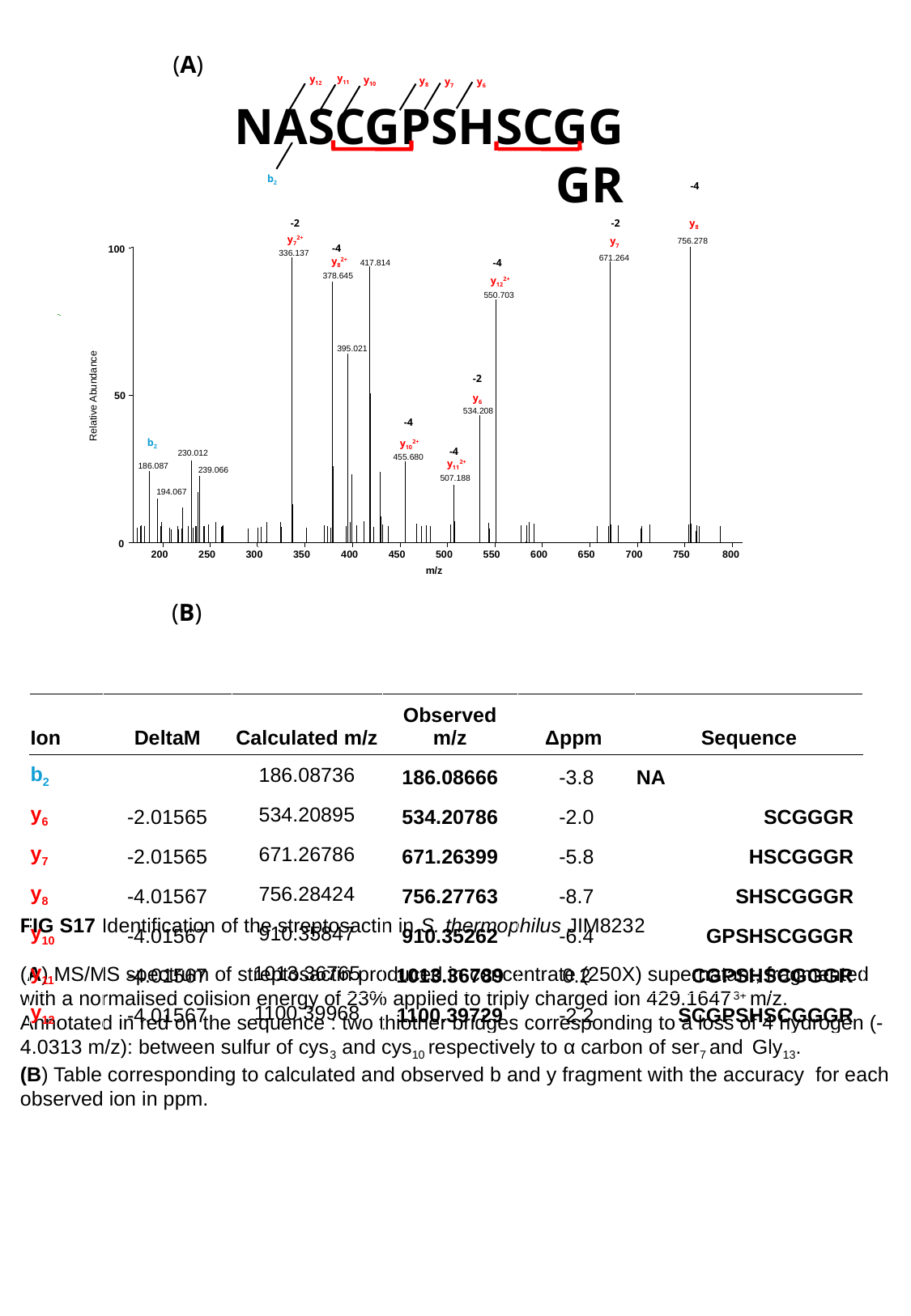

(A)
y11
y12
y10
y8
y7
y6
NASCGPSHSCGGGR
b2
-4
-2
-2
y8
y72+
y7
756.278
-4
100
336.137
y82+
-4
671.264
417.814
y122+
378.645
550.703
395.021
-2
y6
50
Relative Abundance
534.208
-4
b2
y102+
-4
230.012
y112+
455.680
186.087
239.066
507.188
194.067
0
800
200
250
300
350
400
450
500
550
600
650
700
750
m/z
(B)
| Ion | DeltaM | Calculated m/z | Observed m/z | Δppm | Sequence |
| --- | --- | --- | --- | --- | --- |
| b2 | | 186.08736 | 186.08666 | -3.8 | NA |
| y6 | -2.01565 | 534.20895 | 534.20786 | -2.0 | SCGGGR |
| y7 | -2.01565 | 671.26786 | 671.26399 | -5.8 | HSCGGGR |
| y8 | -4.01567 | 756.28424 | 756.27763 | -8.7 | SHSCGGGR |
| y10 | -4.01567 | 910.35847 | 910.35262 | -6.4 | GPSHSCGGGR |
| y11 | -4.01567 | 1013.36765 | 1013.36789 | 0.2 | CGPSHSCGGGR |
| y12 | -4.01567 | 1100.39968 | 1100.39729 | -2.2 | SCGPSHSCGGGR |
FIG S17 Identification of the streptosactin in S. thermophilus JIM8232
(A) MS/MS spectrum of streptosactin produced in concentrate (250X) supernatant, fragmented with a normalised colision energy of 23% applied to triply charged ion 429.16473+ m/z.
Annotated in red on the sequence : two thiother bridges corresponding to a loss of 4 hydrogen (-4.0313 m/z): between sulfur of cys3 and cys10 respectively to α carbon of ser7 and Gly13.
(B) Table corresponding to calculated and observed b and y fragment with the accuracy for each
observed ion in ppm.

## Slide 18
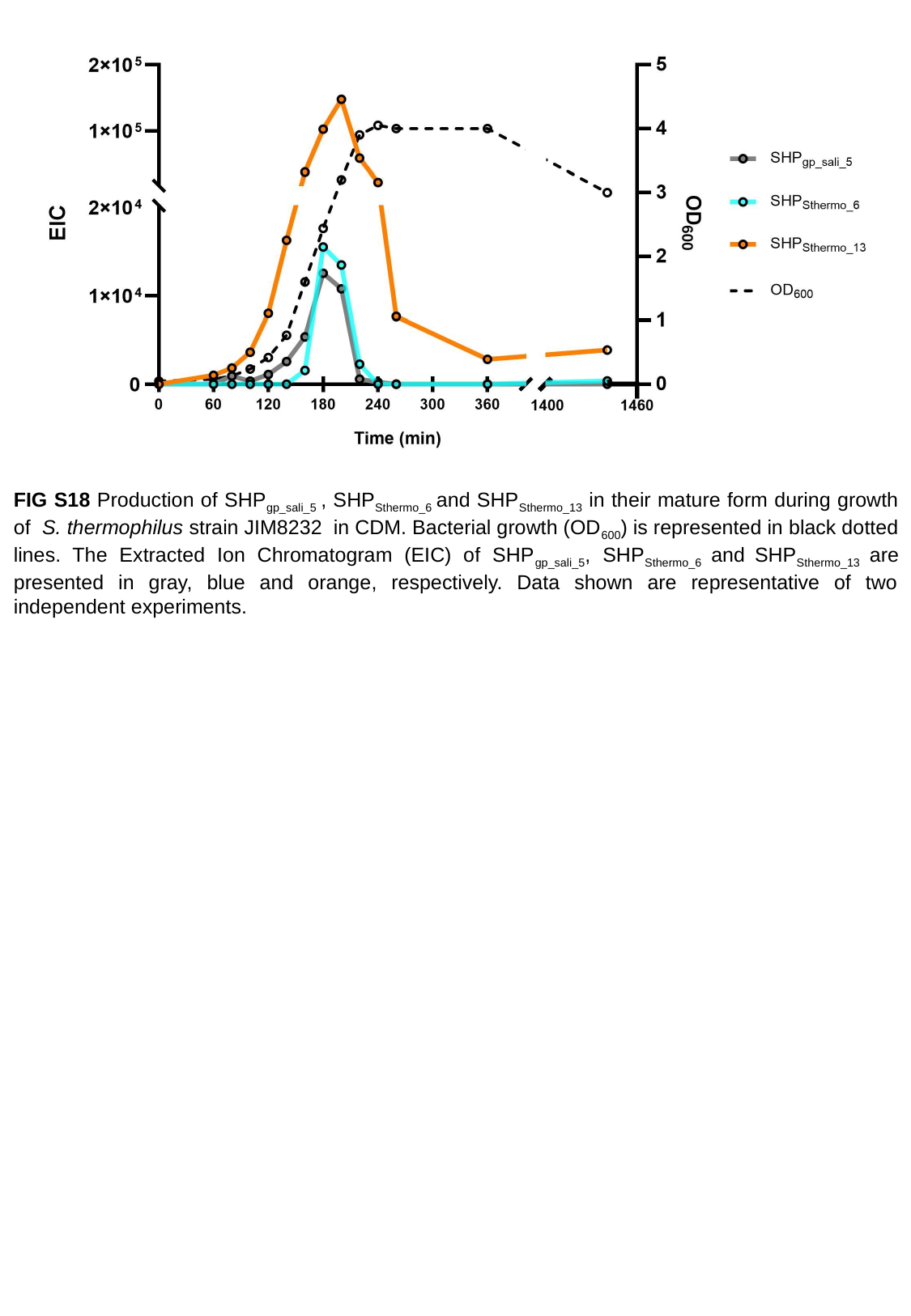

FIG S18 Production of SHPgp_sali_5 , SHPSthermo_6 and SHPSthermo_13 in their mature form during growth of S. thermophilus strain JIM8232 in CDM. Bacterial growth (OD600) is represented in black dotted lines. The Extracted Ion Chromatogram (EIC) of SHPgp_sali_5, SHPSthermo_6 and SHPSthermo_13 are presented in gray, blue and orange, respectively. Data shown are representative of two independent experiments.
