# Supplementary material for: RRNPP quorum-sensing repertoires in the salivarius group genomes: overrepresentation and synchronous activation of SHP/Rgg systems in Streptococcus thermophilus
Source: J Bacteriol. 2025 Aug 26;207(9):e00231-25. doi: 10.1128/jb.00231-25 (PMC12445099; doi:10.1128/jb.00231-25)
Supplement: Supplemental material and methods — Complement to the LC-HR-MS/MS analysis. [file jb.00231-25-s0003.docx]

**Supplementary Material and Methods**

**NanoLC-MS configuration of analysis (NanoRSLC-nanoESI-Tribrid)**

For the detection of all SHP pheromones, 6 µl of supernatants culture were loaded onto a PepMap C18 pre-column (5 mm length x 300 µm I.D., 5 µm particle size, 100 Å, Thermo Fisher Scientific) at a flow rate of 20 µL/min 0.08% TFA in 2% ACN for 2 min. This step was followed by separation on a PepMap Neo C18 column (500 mm length x 75 µm I.D., Thermo Fisher Scientific). The buffers used were 0.1% formic acid in 98% water (Solvent A) and 0.1% formic acid in 80% ACN (Solvent B). The liquid chromatography gradient was linear from 1% to 98% solvent B over 35 minutes at 250 nL/min, with a total run time of 60 min. Full MS scans were automatically calibrated using the lock mass option (445.12002 m/z = polydimethylcyclosiloxanes; 519.13882 m/z; 593.15761 m/z & 667.1764 m/z = polysiloxane). If necessary, retention time was calibrated using riboflavin (377.14611 m/z) as an internal standard of the CDM medium. MS data acquisition included a full MS scan (m/z) range of 300–1800 with a resolution of 15,000. Singly charge SHPs ions (SHP_gp_sali_5_ (900.5401), SHP_Sthermo_6_ (1018.5608), SHP_gp_sali_6_ (898.5608), SHP_gp_sali_7_ (912.57649), SHP_Sthermo_13_ (856.51389), SHP_gp_sali_4_ (897.6020). SHP_gp_sali_3_ (981.50746 carbamidomethylated form), SHP_Sthermo_12_ (799.49242)) were fragmented by tMS² OT HCD method by testing 2 normalized collision energies (18 or 28 at a resolution of 15,000). We choose for each MS/MS peptide analysis, the best fragmented spectrum for a specific normalized collision energy (NCE), for best signal to noise ratio with a maximum number of ions daughter annotated (b and y) and the corresponding retention time with synthetic peptide only for each SHP pheromone. Theses spectra were manually extracted by bases peak MS signal +/- 5 ppm using Xcalibur Qual Browser software with Genesis tools (3.0.63).

**LC and MS configuration for analysis on Vanquish-ESI-Q Exactive Focus:**

For the detection of all interesting peptides in SIM mode with a Q-Exactive Focus mass spectrometer, 10 µl of culture supernatants were loaded onto a Zorbax Eclipse Plus C18 RRHT column (50 mm x 2.1 mm I.D., 1.8 µm particle size, 95 Å, Agilent), with two different flow rate of 0.3 mL/min and 0.45 ml/min. The peptide separation was realized with an acetonitrile gradient in multiple steps. The buffers used were 0.1% formic acid in milliQ water (Solvent A) and 0.1% formic acid in 80% of Acetonitrile (Solvent B):

| Time (min) | Flow (ml/min) | % solvant B |
| --- | --- | --- |
| 0 | 0.3 | 0 |
| 1 | 0.3 | 0 |
| 3.5 | 0.3 | 10 |
| 3.6 | 0.45 | 10 |
| 4 | 0.45 | 20 |
| 9 | 0.45 | 40 |
| 9.2 | 0.45 | 100 |
| 9.5 | 0.45 | 100 |
| 9.8 | 0.45 | 0 |
| 14.5 | 0.3 | 0 |
| 15 | 0.3 | 0 |

Mass spectrometer scans were classically calibrated with Thermo fisher positive calibrant starting the analysis and working during acquisition at a resolution of 70000, with a window of +/- 4 m/z for the selected mass in specific window:

| m/z | time range (min) | Peptides in strain JIM8232 |
| --- | --- | --- |
| 1018.561 | 9-10 | SHP_Sthermo_6_ |
| 899.000 | 7.1-11 | SHP_gp_sali_5_ (900.540) , SHP_gp_sali_6_  (898.561), SHP_gp_sali_4_  (897.602) |
| 856.514 | 6.5-7 | SHP_Sthermo_13_ |
| 307.803 | 0.5-2.5 | bicyclostreptin A (z3) |
| 495.247 | 2.5-5 | streptide (z2) |

The MS signal of singly charge SHPs (SHP_gp_sali_5_, SHP_Sthermo_6_, SHP_gp_sali_6_, SHP_Sthermo_13_ and SHP_gp_sali_4_) and doubly charged ion for streptide and triply m/z for bicyclostreptin A were manually extracted using the same software as above. Finally, we extracted the bases peak MS signal +/- 5 ppm:

SHP_gp_sali_5_ (900.5401), SHP_Sthermo_6_ (1018.5608), SHP_gp_sali_6_ (898.5608), SHP_gp_sali_7_ (912.57649), SHP_Sthermo_13_ (856.51389), SHP_gp_sali_4_ (897.6020~~,~~ streptide (495.247) and for bicyclostreptin A (307.803).For enteropeptin D and streptosactin identification, the gradient was performed with same solvent A and B as above, but with a single step from 0 to 30% Solvent B over 20 min with a flow rate of 0.3 mL/min.
